# (*E*,*Z*)-1,1,1,4,4,4-Hexafluorobut-2-enes: hydrofluoroolefins halogenation/dehydrohalogenation cascade to reach new fluorinated allene

**DOI:** 10.3762/bjoc.20.40

**Published:** 2024-02-27

**Authors:** Nataliia V Kirij, Andrey A Filatov, Yurii L Yagupolskii, Sheng Peng, Lee Sprague

**Affiliations:** 1 Institute of Organic Chemistry, National Academy of Sciences of Ukraine, Academician Kukhar Str., 5, Kyiv-94, 02660, Ukraine; 2 The Chemours Company, Chemours Discovery Hub, Newark, DE 19713, United States

**Keywords:** allenes, dehydrohalogenation, halogenation, 1,1,1,4,4,4-hexafluorobut-2-enes, isomerization

## Abstract

A series of 2,3-dihalo-1,1,1,4,4,4-hexafluorobutanes and 2-halo-1,1,1,4,4,4-hexafluorobut-2-enes were prepared from commercially available hydrofluoroolefins 1,1,1,4,4,4-hexafluorobut-2-enes and their ^1^H, ^19^F and ^13^C chemical shifts measured. Some reactions of synthesized 2-halo-1,1,1,4,4,4-hexafluorobut-2-enes have been investigated. A simple, one-pot procedure for the preparation of a new allene (1,1,4,4,4-pentafluorobuta-1,2-diene) and some of its transformations is presented.

## Introduction

The first publication using (*E*)-1,1,1,4,4,4-hexafluorobut-2-ene (**1a**) was published in the middle of the 20th century [1]. Despite this, the real opportunity to study the properties of 1,1,1,4,4,4-hexafluorobut-2-ene appeared only after (*Z*)-1,1,1,4,4,4-hexafluorobut-2-ene (**1b**) began to be produced on an industrial scale [2]. These hydrofluoroolefins belong to the newest 4th generation of fluorocarbon refrigerants and are promising compounds and starting materials. Due to this, interest in the use of (*E*)- and (*Z*)-butenes **1a**,**b** as synthons in various organic transformations has recently grown significantly. One of the new and budding directions in recent years is the stereoselective olefin metathesis processes based on catalysis by complexes of molybdenum, tungsten and ruthenium [3–5]. The first publications have recently appeared that molybdenum complexes can catalyze cross-metathesis of butene **1b**. Wherein various alkyl and aryl olefins, including those that contain Lewis basic esters, carbamates and amines or α-branched moieties, may be used in efficient and exceptionally Z-selective cross-metathesis reactions [6–8]. A few years ago, some publications devoted to the cleavage of the C–F bond in butenes **1a**,**b** have been presented in the literature. First, Crimmin et al. investigated the reaction of an aluminum(I) complex with fluoroalkenes. Unlike all the presented fluoroolefins, the reaction of the Al(I) complex with (*Z*)-butene **1b** did not allow isolating the intermediate organoaluminum compound, but led to the elimination of two fluorine atoms with the formation of 1,1,4,4-tetrafluorobuta-1,3-diene [9]. It was also shown that the reactions of the boron reagent (CAACMe)BH_2_Li(thf)_3_ with hydrofluoroolefins, including (*Z*)-1,1,1,4,4,4-hexafluorobut-2-ene, results in defluoroborylation to form the corresponding =CF_2_ containing products [10]. In addition to complexes of aluminum and boron, several magnesium and lithium silyl reagents were prepared and proved to be good nucleophiles in reactions with (*Z*)-1,1,1,4,4,4-hexafluorobut-2-ene, as a result of which the corresponding defluorosilylation product was obtained [11]. In a related study of the hydrosilylation reaction of olefins **1a**,**b**, it was shown that, depending on the catalyst used, platinum or rhodium compounds, along with the products of the addition of silane to the double bond, the elimination of the fluorine atom occurs with the formation of the corresponding olefin [12]. Another area of application of olefins **1a,b** is based on C=C double bond addition reactions. As early as 1968, Atherton and Fields showed that (*Z*)- and (*E*)-butenes **1a**,**b** reacted with diazotrifluoroethane to give 3,4,5-tris(trifluoromethyl)pyrazoline [13]. A few decades later, several new publications in this direction appeared in the literature. In a Chemours’ patent, it has been shown that the oxidation of (*E*)-butene **1a** with sodium hypochlorite in the presence of tetrabutylphosphonium bromide leads to the formation of a bistrifluoromethyl containing oxirane [14]. A related study has demonstrated that (*E*)-butene **1a** reacts with potassium persulfate to form 4,5-bistrifluoromethyl)-1,3,2-dioxathiolane 2,2-dioxide [15]. In 2021 Petrov published an article on the interaction of fluorinated olefins with fluorinated thioketones. In this publication it was demonstrated that 1,1,1,4,4,4-hexafluorobut-2-ene reacts with dithietane, sulfur and KF with the formation of the corresponding 1,3-dithiole [16]. Also, a recent patent presents a method for the preparation of 5,6-bis(trifluoromethyl)-1,2,4-triazine-3-carboxylic acid ethyl ester starting from 1,1,1,4,4,4-hexafluorobut-2-ene and an oxalamide hydrazone [17].

In the present study, we investigated the reactions of commercially available butenes **1a**,**b** with halogens, as well as subsequent transformations of the resulting compounds.

## Results and Discussion

In 1952 Haszeldine found that the reaction of bromine with (*E*)-1,1,1,4,4,4-hexafluorobut-2-ene (**1a**) under UV irradiation leads to the formation of 2,3-dibromo-1,1,1,4,4,4-hexafluorobutane (**2**) [1]. Subsequent dehydrobromination of compound **2** by treatment with alcoholic potassium hydroxide formed a mixture of isomers 2-bromo-1,1,1,4,4,4-hexafluorobut-2-ene (**3a**,**b**) with a yield in two stages of 48%. It should be noted, that only boiling points and elemental analysis data were given for the obtained substances. Ten years later Knunyants and co-workers also synthesized compound **2** and density, refractive index and mass spectra were added to the already available data [18]. However, until now there was no information about the structure and spectral characteristics of the obtained compounds. We have now synthesized these compounds, fully characterized them, and studied some of their transformations.

**Scheme 1:**
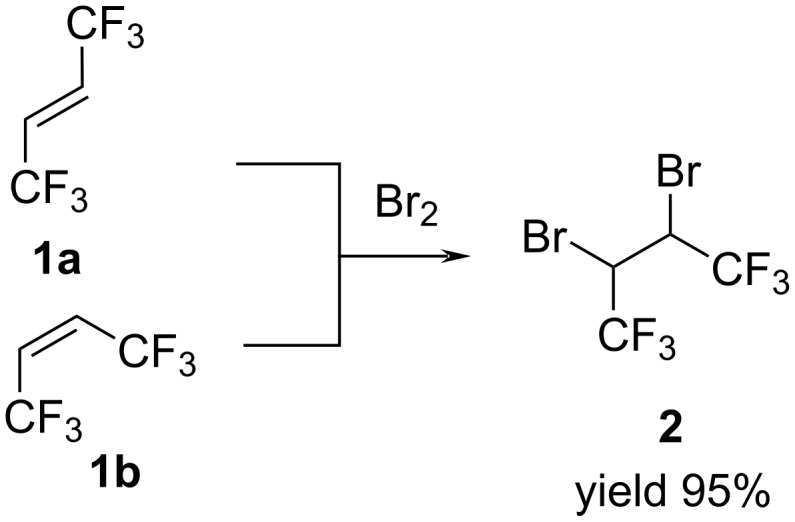
Synthesis of 2,3-dibromo-1,1,1,4,4,4-hexafluorobutane (**2**).

We found that not only (*E*)-butene **1a** but also (*Z*)-butene **1b** reacted with bromine in the same manner under the influence of ultraviolet irradiation or sunlight with the formation of 2,3-dibromo-1,1,1,4,4,4-hexafluorobutane (**2**) in 95% yield (Scheme 1).

The only difference was that under UV irradiation, the reaction proceeded faster. In both cases, product **2** represented a mixture of stereoisomers in 2:1 ratio. After isolation by distillation, 2,3-dibromo-1,1,1,4,4,4-hexafluorobutane (**2**) was characterized by ^1^H, ^19^F, ^13^C NMR and mass spectra.

We studied the reaction of dibromoalkane **2** with various bases such as DBU, Hünig’s base (iPr_2_NEt), and potassium hydroxide (Table 1).

**Table 1:** The reaction of 2,3-dibromo-1,1,1,4,4,4-hexafluorobutane (**2**) with bases.

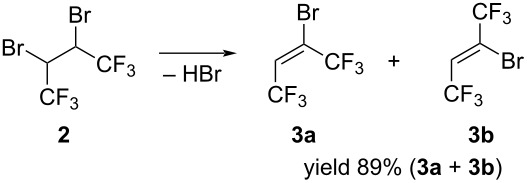				

Entry	Solvent	Base (equiv)	Conditions (°C/h)	Conversion (%)

1	Et_2_O	iPr_2_NEt (1)	20/18	60
2	Et_2_O	iPr_2_NEt (1)	20/18	95
3	Et_2_O	DBU (1)	20/1	95
4	diglyme	DBU (1)	20/18	60
5	diglyme	DBU (2)	20/1	100
6	H_2_O	KOH (1)	20/18	55
7	H_2_O	KOH (1.3)	20/2	100

In all cases, except the reaction in diglyme (Table 1, entry 5), a mixture of (*E*)- and (*Z*)-2-bromo-1,1,1,4,4,4-hexafluorobut-2-enes (**3a**,**b**) in a ratio of 2:1 was formed. The configuration of the isomers was determined by the ^5^*J*_FFcis_ coupling constant in the ^19^F{H} NMR spectrum (ca. 0 Hz for (*Z*)-isomer and ca. 11 Hz for (*E*)-isomer). The best results were obtained in Et_2_O with Hünig’s and DBU bases (Table 1, entries 2 and 3), but unfortunately in these cases the product olefins could not be separated from Et_2_O. Therefore, we decided to use high-boiling diglyme instead of ether. The reaction of a butane **2** with one equivalent of DBU (Table 1, entry 4) led to the same results as for the Hünig’s base (Table 1, entry 1). The use of two equivalents of DBU (Table 1, entry 6) led to the complete conversion of the initial substrate, but the selectivity of the reaction was significantly reduced in this case. The reaction mixture gave a complex mixture, in which (*E*)-butene **3a** was identified as a major component. Product **3a** was removed from bulk diglyme in vacuum and after subsequent distillation it was isolated with a yield of 23%. As the isolation of the 2-dehydrobromination products from organic bases was complex, we focused our attention on carrying out the reaction with KOH. The best result was obtained by treatment of 1 equivalent of butane **2** with 1.3 equivalents of KOH in the presence of 5 mol % of Bu_4_NBr as an interfacial carrier in water at room temperature for 2 h (Table 1, entry 7). After completion of the reaction the mixture of isomers **3a**,**b** was separated from the water phase and distilled at 55 °C.

**Scheme 2:**
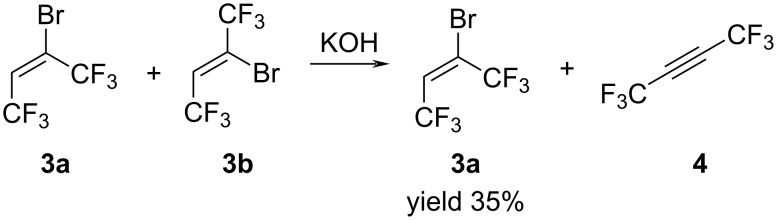
Synthesis of (*E*)-butene **3a**.

Further increase in the amount of KOH led to the elimination of the second mole of HBr with the formation of hexafluorobut-2-yne (**4**). By controlling the course of the reaction by the ^19^F NMR method it was possible to achieve complete dehydrobromination of (*Z*)-isomer **3b** and isolation of (*E*)-isomer **3a** in pure form in 35% yield (Scheme 2).

**Scheme 3:**
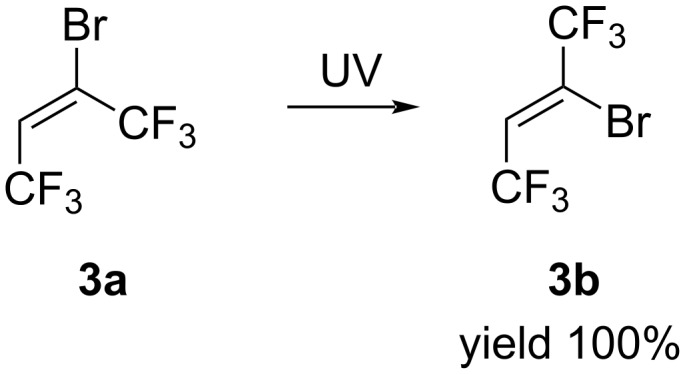
Isomerization reaction of (*E*)-butene **3a** to (*Z*)-butene **3b**.

Unexpectedly the (*E*)-isomer **3a**, upon long-term storage, transforms into the (*Z*)-isomer **3b**. Therefore, we studied the isomerization of olefin **3a** in the presence of SbCl_5_, AlCl_3_, Bu_4_NOH/MeOH and under UV irradiation. We found that under UV irradiation for several hours, the (*E*)-isomer **3a** completely transformed into (*Z*)-isomer **3b** in quantitative yield (Scheme 3).

**Scheme 4:**
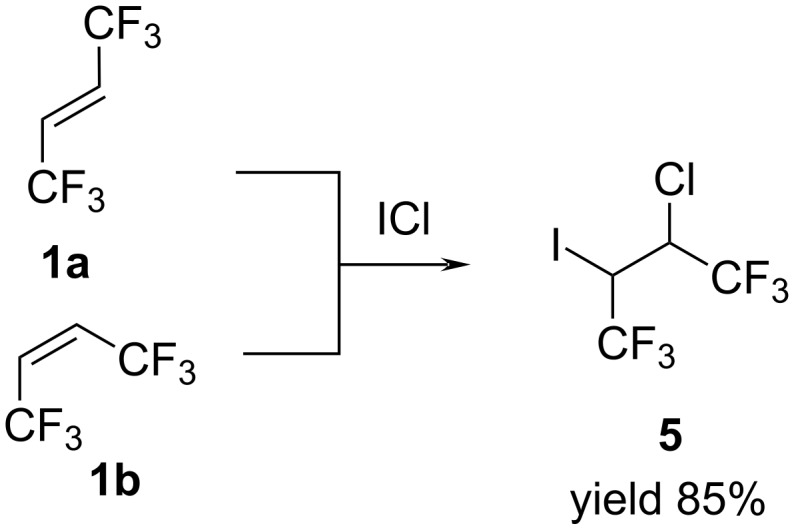
Synthesis of 2-chloro-3-iodo-1,1,1,4,4,4-hexafluorobutane (**5**).

Next, our attention was directed toward the reaction of (*E*)- and (*Z*)-butenes **1a**,**b** with iodine monochloride (ICl). We found that olefins **1a** and **1b** reacts with ICl under the influence of sunlight to form previously unknown 2-chloro-3-iodo-1,1,1,4,4,4-hexafluorobutane (**5**, Scheme 4).

Pure final product **5** was isolated by distillation at 112 °C in 85% yield. NMR analysis as in the case of bromo derivative **2** showed a mixture of stereoisomers with a 2:1 ratio.

**Scheme 5:**
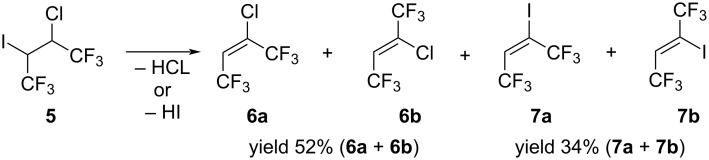
Dehydrohalogenation reaction of 2-chloro-3-iodo-1,1,1,4,4,4-hexafluorobutane (**5**).

The dehydrohalogenation reaction of 2-chloro-3-iodo-1,1,1,4,4,4-hexafluorobutane (**5**) was studied. Like the dehydrobromination of alkane **2**, the reaction of compound **5** with 1.3 equivalents of KOH in water in the presence of 5 mol % of Bu_4_NBr was carried out. In this case, a mixture of 2-chloro-1,1,1,4,4,4-hexafluorobut-2-enes (**6a**,**b**) and 2-iodo-1,1,1,4,4,4-hexafluorobut-2-enes (**7a**,**b**) in a ratio of 3:2 was formed from the concurrent elimination reactions of hydrogen iodide and hydrogen chloride (Scheme 5).

The chloro- and iodobutenes were separated by distillation in 52% yield for **6a**,**b** and 34% yield for **7a**,**b**. NMR analysis showed the mixtures of (*E*)- and (*Z*)-isomers in both cases with the ratio *E*/*Z* = ≈4:5 for **6a**,**b** and *E*/*Z* = ≈1:2 for **7a**,**b**. The configuration of the isomers was determined by the ^5^*J*_FFcis_ coupling constant in the ^19^F{H} NMR spectrum (ca. 0 Hz for (*Z*)-isomer and ≈11 Hz for (*E*)-isomer). The 2-chloro-1,1,1,4,4,4-hexafluorobut-2-enes were first described by Haszeldine in 1952 [1] and only then the (*Z*)-isomer. We report here the isolation and complete characterization of both (*E*)- and (*Z*)-isomers. The spectral characteristics of product **6b** obtained by us fully correspond to the literature data [19–20]. The ^1^H and ^19^F NMR spectra of compounds **7a**,**b** also corresponded to the data given in the literature [21–22]. We present here the spectral data for isomer **6a**, as well as the missing data of ^13^C NMR spectra for iodoolefins **7a**,**b**.

It should be noted that the reaction of alkane **5** with DBU or Hünig’s base in Et_2_O or dimethoxyethane (DME) as a solvent, resulted only in the formation of 2-chloro-1,1,1,4,4,4-hexafluorobut-2-enes **6a**,**b** (Scheme 5). Unfortunately, due to difficulties in separation from solvent, olefins **6a**,**b** in this case were not isolated in a pure state.

**Scheme 6:**
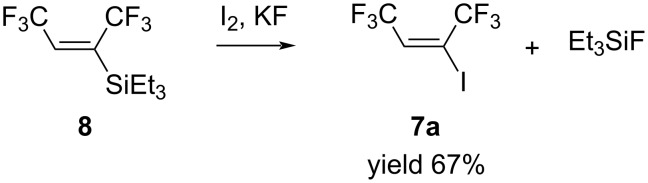
The reaction of silane **8** with I_2_/KF.

For iodoolefin **7a**, we found an alternative route for its synthesis. We have previously shown that hydrosilylation reaction of hexafluorobut-2-yne with triethylsilanes gave (*E*)-1,1,1,4,4,4-hexafluoro-2-triethylsilylbut-2-ene (**8**) [23]. Going back to the study of the obtained silane reactivity we performed the reaction with iodine. All experiments were carried out in THF, *N*-methylpyrrolidone and sulfolane with iodine in the presence of a source of fluoride ion. The best result was observed when the reaction was carried out in dry sulfolane with a two-fold excess of iodine and 1.5-fold excess of anhydrous KF (Scheme 6).

The mixture was stirred at 30 °C for several days until none of the starting **8** was detected in ^19^F NMR spectra. The desired iodoolefin **7a** together with byproduct triethylfluorosilane (Et_3_SiF) were removed from sulfolane under vacuum and after double distillation with column **7a** was isolated in 67% yield. The ^19^F{1H} NMR spectra of **7a**, showed the coupling constant of CF_3_ and CF_3_ to be 11.3 Hz, suggesting strongly that product **7a** like the original silane **8** has (*E*)-configuration.

Although some olefins presented above were obtained several decades ago, there was almost no information about their reactivity in the literature. Therefore, we became interested in exploring the synthetic potential of halo-bistrifluoromethyl-containing olefins **3**, **6** and **7**, which are readily available and can be synthesized in the laboratory in appreciable amounts.

Fluorinated organic compounds are important synthons, which are widely used in agrochemicals, pharmaceuticals and other fields [24–26]. Fluoroorganic lithium and Grignard reagents have been obtained by the metalation reactions of organofluorine compounds containing bromine and iodine atoms with alkyllithium and Grignard reagents.

Although olefin **3** has been available for many decades, only one paper describes its lithiation with methyllithium and the subsequent reaction of the lithium compound with trifluoroacetophenone [27]. We began our research on the reactivity of the bromobutenes **3a**,**b** with isopropylmagnesium chloride (iPrMgCl) and butyllithium (BuLi), as well as the reactions of the resulting organometallic compounds.

**Scheme 7:**
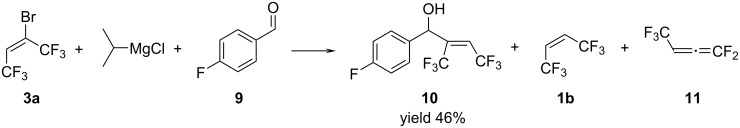
The reaction of **3a** with iPrMgCl and 4-fluorobenzaldehyde (**9**).

1.2 Equivalents of a solution of iPrMgCl in THF were added to bromoolefin **3a** in Et_2_O or THF at −78 °C and then after 1 h at the same temperature 1 equivalent of 4-fluorobenzaldehyde (**9**) was added. After completion of the reaction and subsequent treatment of the reaction mixture with 2 N hydrochloric acid, 2,3-bis(trifluoromethyl)-1-(4-fluorophenyl)prop-2-ene-1-ol (**10**) was detected in the ^19^F NMR spectrum. It should be noted that, in addition to the unreacted starting aldehyde **9**, the formation of olefin **1b** and previously unknown product **11** were also recorded in the reaction mixture (Scheme 7). The ^19^F NMR spectrum of compound **11** showed two signals at −65.6 and −99.1 ppm in a ratio of 3:2 and in the ^1^H NMR spectrum, a multiplet at 6.5 ppm was detected. Based on the received data, we assumed that product **11** had an allene structure. It was also important to note that the reaction proceeded more selectively in ether, which significantly reduced the amount of byproducts.

Pure final alcohol **10** was isolated by column chromatography on SiO_2_ in 46% yield and ^1^H, ^19^F and ^13^C NMR spectra were in full agreement with the published data [23].

The most interesting outcome from our point of view was the formation of 1,1,4,4,4-pentafluorobuta-1,2-diene (**11**). 1,1-Difluoroallenes are building blocks for a great number of valuable transformations [28]. Therefore, the synthesis of new fluorinated allenes continues to be relevant. One of the methods for the synthesis of allenes was based on the interaction of bromoolefins with organolithium compounds, followed by the elimination of lithium fluoride [29–31]. It was logical to assume that in our case a similar reaction of the Grignard reagent **12** with aldehyde **9**, elimination of MgBrF results in the formation of allene **11**. To confirm our hypothesis, we studied the reaction of haloolefins **3** and **7** with iPrMgCl and BuLi.

**Scheme 8:**
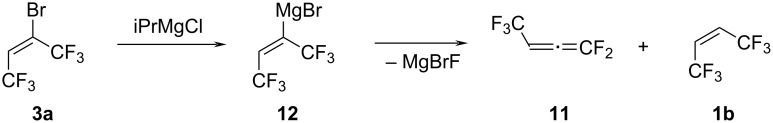
The reaction of olefin **3a** with iPrMgCl.

Thus, olefin **3a** in Et_2_O reacted with iPrMgCl solution in THF at −80 °C to form Grignard reagent **12** and by heating the reaction mixture to room temperature MgBrF was produced together with allene **11** (Scheme 8). In addition to the allene, the formation of olefin **1b** was also recorded in the ^19^F NMR spectrum.

Unfortunately, due to difficulties in separating from olefin **1b** and diethyl ether, allene **11** was not isolated in a pure state. Therefore, we turned to study the reaction of adduct **3a** with butyllithium.

**Scheme 9:**
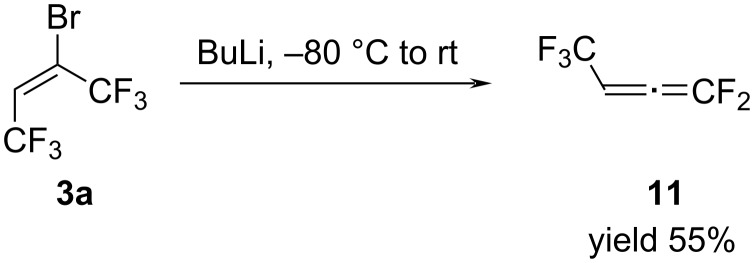
The reaction of (*E*)-butene **3a** with BuLi.

Unlike the reaction of bromobutene **3a** with iPrMgCl, the reaction mixture with BuLi in hexane solution did not contain olefin **1b** and only desired allene **11** was identified with 95% purity in the ^19^F NMR spectra (Scheme 9).

The volatile product **11** was removed from the reaction mixture at 20 °C for 2 hours with a slow flow of argon through the system. Unfortunately, we failed to completely separate it from the hexane and allene **11** was produced with 80–85% purity. We managed to partially solve this problem by replacing hexane with the higher-boiling heptane. In this case, the desired product was isolated by double distillation at 7 °C in 50% yield and >90% purity and was fully characterized by ^1^H, ^19^F, ^13^C NMR and IR spectra. The presence of a characteristic band at 2038 cm^−1^ in the IR spectrum confirmed the allene structure of product **11**. As expected, allene **11** is extremely reactive and therefore it decomposes rather quickly during storage both in solvent and in the individual state.

Since we were able to obtain both isomers **3a** and **3b** in the individual state, we investigated the reaction of (*Z*)-isomer **3b** with BuLi. Thus, **3b** also reacted with butyllithium to form allene **11**. However, when studying the reaction of each of the isomers under the same conditions, it turned out that the (*Z*)-isomer reacts faster than the (*E*)-isomer. Therefore, in the case of the (*E*)-isomer, to achieve its complete conversion, it was necessary to increase the lithiation reaction time. Based on the results obtained, it was logical to assume that the reaction can be carried out with a mixture of **3a**,**b**. We found that the reaction of **3a**,**b** with 1.2 equivalents of BuLi in heptane at −80 °C for 1 hour, followed by warming to room temperature, led to the complete conversion of the original olefins and the formation of allene **11**.

Thus, we have developed a method for the synthesis of the previously unknown allene **11**. In addition, the possibility of using a mixture of olefins **3a**,**b** made the allene **11** a more accessible synthon for studying its further transformations.

**Scheme 10:**
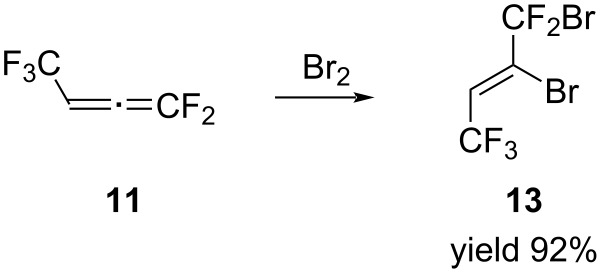
The reaction of allene **11** with bromine.

We started studying the reactivity of allene **11** with the bromination reaction. The reaction was carried out without a solvent at a reagent ratio of 1:1. Bromine was added dropwise to allene **11** at −30 °C and the reaction mixture was stirred at the same temperature until colorless. The bromination of allene resulted in the formation of (*Z*)-1,2-dibromo-1,1,4,4,4-pentafluorobut-2-ene (**13**) in 92% yield, which was isolated as a colorless liquid and fully characterized by NMR spectroscopy (Scheme 10).

The ^19^F NMR spectrum showed two signals with the integrated intensity ratio 3:2, a doublet for a CF_3_ group at δ −57.7 ppm with the coupling constant ^3^*J*_FH_ = 6.8 Hz and the characteristic signal of a CF_2_Br group as a singlet at δ −50.1 ppm. In the ^1^H NMR spectrum, there was a signal attributed to the CH proton at 6.9 ppm as a quartet with the coupling constant of ^3^*J*_HF_ = 6.8 Hz. The (*Z*)-configuration of product **13** was determined by the absence of the F–F constant in the ^19^F NMR spectra. It should be noted that the bromination reaction could also be carried out in diethyl ether. In this case, the bromo derivative **13** could be isolated in pure form only after several distillations, which significantly reduces its yield.

**Scheme 11:**
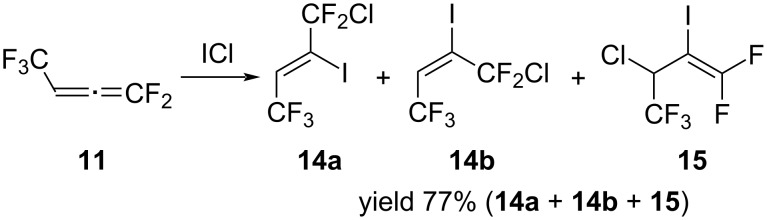
The reaction of allene **11** with ICl.

Continuing to study the reactivity of allene **11**, we investigated its reaction with ICl. The reaction was carried out at a reagent ratio of 1:1. ICl was added to a solution of allene in pentane at −20 °C and then the reaction mixture was stirred at rt until the color disappeared. In contrast to bromination, the reaction with ICl is less selective and leads to the formation of addition products at both double bonds. At the same time, the reaction proceeded predominantly at the terminal double bond with the formation of a mixture of isomers (*Z*)-**14a** and (*E*)-**14b**. The content of the addition product **15** at the second double bond in the reaction mixture was about 15% (Scheme 11).

It should be noted that we failed to isolate the pure product **15** from reaction mixtures because of the small difference in boiling points and volatiles between isomers **14a**,**b** and product **15**. Nevertheless, it was tentatively identified in the mixture, based on ^1^H, ^19^F and ^13^C NMR data. The ^19^F NMR spectrum showed three signals with the integrated intensity ratio 3:1:1, a doublet for a CF_3_ group at δ −71.5 ppm with a coupling constant of ^3^*J*_FH_ = 6 Hz and two highly characteristic signals of geminal fluorine atoms of the CF_2_ group as doublets of multiplets at δ −66.7 and −70.3 ppm with a coupling constant of ^2^*J*_FF_ = 15 Hz. In the ^1^H NMR spectrum, there was a signal attributed to CH proton at 4.9 ppm as a quartet of multiplets with a coupling constant of ^3^*J*_HF_ = 6 Hz.

Thus, during the study of reactions of allene **11** with Br_2_ and ICl, we obtained new synthons, which due to the presence of several reaction centers, could be of particular interest in various kinds of transformations. Therefore, we decided to synthesize a bistrifluoromethyl containing olefin with bromine and chlorine atoms and explore the possibility of using it to obtain another allene.

**Scheme 12:**
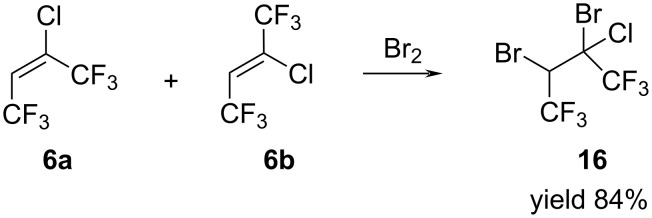
Synthesis of 2,3-dibromo-2-chloro-1,1,1,4,4,4-hexafluorobutane (**16**).

We found that 2-chloro-1,1,1,4,4,4-hexafluorobut-2-enes (**6a**,**b**) react with bromine under the influence of sunlight with the formation of 2,3-dibromo-2-chloro-1,1,1,4,4,4-hexafluorobutane **16** in 84% yield (Scheme 12).

As in the case of alkanes **2** and **5**, product **16** represented a mixture of stereoisomers in 2:1 ratio. After isolation by distillation butane **16** was characterized by ^1^H, ^19^F, ^13^C NMR and mass spectra.

**Scheme 13:**
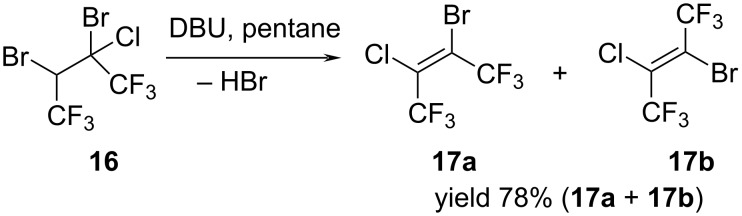
Synthesis of (*Z*, *E*)-2-bromo-3-chloro-1,1,1,4,4,4-hexafluorobut-2-enes (**17a**,**b**).

The reaction of alkane **16** with DBU in pentane as a solvent led exclusively to dehydrobromination with the formation of a mixture of (*Z*)- and (*E*)-2-bromo-3-chloro-1,1,1,4,4,4-hexafluorobut-2-enes (**17a**,**b**) in a ratio of 2:1 (Scheme 13).

After completion of the reaction the mixture of isomers **17a**,**b** was isolated by distillation at 79 °C and fully characterized. The configuration of the isomers was determined by the ^5^*J*_FFcis_ coupling constant in the ^19^F{H} NMR spectrum (ca. 0 Hz for (*Z*)-isomer and ca. 13 Hz for (*E*)-isomer).

**Scheme 14:**
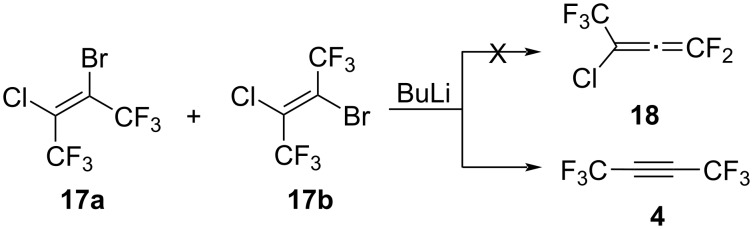
The reaction of olefins **17a**,**b** with BuLi.

Like the reaction of bromobutenes **3a**,**b** with BuLi, the reaction of olefins **17a**,**b** with BuLi was carried out. Unfortunately, we were unable to detect the formation of allene **18** in this reaction. In the ^19^F NMR spectrum, along with the unreacted starting olefins **17a**,**b**, the main reaction product is hexafluorobut-2-yne (**4**, Scheme 14).

Although attempts to obtain allene **18** were unsuccessful, olefins **17a**,**b** may be of great interest as synthons due to the presence of several reaction centers in the molecule.

## Conclusion

In conclusion, we synthesized a series of synthons from available industrial starting compounds – (*E*,*Z*)-1,1,1,4,4,4-hexafluorobut-2-enes (**1a**,**b**) using simple procedures. We demonstrated the halogenation reactions of butenes **1a**,**b** with bromine and iodine monochloride to form 2,3-dihalo-1,1,1,4,4,4-hexafluorobutanes. Dehydrohalogenation of the obtained butanes leads to the formation of a number of bistrifluoromethyl-containing haloolefins, which are widely used in subsequent transformations. Based on bromoolefins **3a**,**b**, a new polyfluoro-containing allene **11** was synthesized and its reactions with bromine and iodine monochloride were also studied.

## Supporting Information

File 1 Experimental part and copies of NMR spectra.
