# Supplementary material for: (E,Z)-1,1,1,4,4,4-Hexafluorobut-2-enes: hydrofluoroolefins halogenation/dehydrohalogenation cascade to reach new fluorinated allene
Source: Beilstein J Org Chem. 2024 Feb 27;20:452–9. doi: 10.3762/bjoc.20.40 (PMC10910381; doi:10.3762/bjoc.20.40)
Supplement: File 1 — Experimental part and copies of NMR spectra. [file Beilstein_J_Org_Chem-20-452-s001.pdf]

BEILSTEIN JOURNAL OF ORGANIC CHEMISTRY

Supporting Information

for

(*E*,*Z*)-1,1,1,4,4,4-Hexafluorobut-2-enes: hydrofluoroolefins halogenation/dehydrohalogenation cascade to reach new fluorinated allene

Nataliia V. Kirij, Andrey A. Filatov, Yurii L. Yagupolskii, Sheng Peng and Lee Sprague

Beilstein J. Org. Chem. **2024,** *20,* 452–459. doi:10.3762/bjoc.20.40

Experimental part and copies of NMR spectra

License and Terms: This is a supporting information file under the terms of the Creative Commons Attribution License (https://creativecommons.org/licenses/by/4.0). Please note that the reuse, redistribution and reproduction in particular requires that the author(s) and source are credited and that individual graphics may be subject to special legal provisions.
The license is subject to the *Beilstein Journal of Organic Chemistry* terms and conditions: (https://www.beilstein-journals.org/bjoc/terms)

Content:

1. Methods S3–10

2. Copies of NMR, IR and mass-spectra of compounds S11–34

3. References S35

1. Methods:

Synthesis

General information: Reactions were carried out under dry argon using flame-dried glassware. DME and Et_2O were distilled over sodium hydride. Pentane, hexane and heptane were distilled over P_2O_5 before use. (*E*,*Z*)-1,1,1,4,4,4- hexafluorobut-2-ene (**1a,b**) were provided by the Chemours' company.

^{1}H NMR spectra were recorded at 400 MHz with a Varian UNITY – Plus 400 spectrometer or at 301.5 MHz with a Mercury + 300 Varian spectrometer. ^{19}F NMR spectra were recorded with a Varian Geminy-200 spectrometer at 188.14 MHz or a Varian UNITY – Plus 400 spectrometer at 376.5 MHz. ^{13}C NMR spectra were recorded on a Bruker AVANCE DRX 500 spectrometer at 125.67 MHz or with an Agilent ProPulse 600 spectrometer at 150.8 MHz. Chemical shifts are given relative to Me_4Si and CCl_3F as internal standards. GC–MS spectra were registered on a Hewlett-Packard HP GC/MS 58,905,972 instrument (EI 70 eV) (Philips, Bothell, WA, USA). IR spectra were recorded on a VERTER 70 spectrometer. Flash chromatography was performed using Merck silica gel 60 (0.063–0.200 mm).

Preparation of 2,3-dibromo-1,1,1,4,4,4-hexafluorobutane (2)

(*E*)- or (*Z*)-1,1,1,4,4,4-hexafluorobut-2-enes (**1a**,**b**) (16.4 g, 0.1 mol) and bromine (16 g, 0.1 mol) stirred under exposure to ultraviolet irradiation or sunlight until the color disappears. The reaction mixture was washed with saturated aqueous solution of Na_2SO_3 (50 mL) to remove bromine residues, dried with $MgSO_4$ and distilled at 116 °C (750 mmHg) affording pure slightly yellowish product **2**; yield 30.76 g (95%). ^{1}H NMR (301.5 MHz, $CDCl_3$): δ = 4.61 (m, 2H); ^{19}F NMR (188.14 MHz, $CDCl_3$): δ = –67.13 (m, 6F, isomer-1), –70.52 (m, 6F, isomer-2); ^{13}C NMR (150.8 MHz, $CDCl_3$): δ = 42.65 (q, $^2J_{CF}$ = 33.9 Hz), 43.57 (q, $^2J_{CF}$ = 37.7 Hz), 121.85 (q, $^1J_{CF}$ = 281.6 Hz),

122.12 (q, $^1J_{CF}$ = 279.1 Hz); GCMS *m/z*: 321.9/323.9/325/9 $[M]^+$, 252.8/254.8/256.8 $[M-CF_3]^+$, 242.9/244.9 $[M-Br]^+$, 163.9 $[M-Br_2]^+$, 69 $[CF_3]^+$.

Dehydrobromination of 2,3-dibromo-1,1,1,4,4,4-hexafluorobutane (2)

To butane **2** (32.4 g, 0.1 mol) suspended in 70 mL of water were added Bu_4NBr (1.61 g, 5 mmol) and a solution of KOH (7.28 g, 0.13 mol) in 35 mL of water. The reaction mixture was stirred at room temperature (completion of the reaction was monitored by ^{19}F NMR spectra). The crude product was separated from the aqueous phase, dried with $MgSO_4$ and distilled to give the mixture of (*E*)- and (*Z*)-2-bromo-1,1,1,4,4,4-hexafluorobut-2-enes (**3a**,**b**) in a ratio of 2:1 according to the NMR spectra; b.p. 55 °C (750 mm Hg), yield 21.62 g (89%). ^{1}H NMR (400 MHz, $CDCl_3$): δ = 6.61 (q, $^3J_{HF}$ = 8 Hz, 1H, *E*-isomer), 7.09 (q, $^3J_{HF}$ = 6.4 Hz, 1H, *Z*-isomer); ^{19}F NMR (376.5 MHz, $CDCl_3$): δ = –58.59 (qd, $^5J_{FF}$ = 11.3 Hz, $^3J_{FH}$ = 8 Hz, 3F, *E*-isomer), –63.67 (q, $^5J_{FF}$ = 11.3 Hz, 3F, *E*-isomer), –62.0 (d, $^3J_{FH}$ = 6.4 Hz, 3F, *Z*-isomer), –69.59 (s, 3F, *Z*-isomer). GCMS *m/z*: 242.9 $[M]^+$, 172.8/174.8 $[M-CF_3H]^+$, 160.9/162.9 $[M-HBr]^+$, 69 $[CF_3]^+$.

Preparation of (*E*)-2-bromo-1,1,1,4,4,4-hexafluorobut-2-ene (3a)

According to the procedure for dehydrobromination of 2,3-dibromo-1,1,1,4,4,4-hexafluorobutane (**2**) the reaction of a mixture of isomers **3a**,**b** (24.29 g, 0.1 mol) suspended in 50 mL of water with Bu_4NBr (1.61 g, 5 mmol) and a solution of KOH (5.6 g, 0.1 mol) in 25 mL of water was added. The reaction mixture was stirred at room temperature (completion of the reaction was monitored by ^{19}F NMR spectra). (*E*)-2-Bromo-1,1,1,4,4,4-hexafluorobut-2-ene (**3a**) was separated from the aqueous phase, dried with $MgSO_4$ and distilled, b.p. 59 °C (750 mmHg), yield 8.5 g (35%). ^{1}H NMR (400 MHz, $CDCl_3$): δ = 6.69 (q, $^3J_{HF}$ = 8 Hz, 1H); ^{19}F NMR (376.5 MHz, $CDCl_3$): δ = –58.21 (qd, $^5J_{FF}$ = 11.3 Hz, $^3J_{FH}$ = 8 Hz, 3F), –63.54 (q, $^5J_{FF}$ = 11.3 Hz, 3F); ^{13}C NMR (125.67 MHz, $CDCl_3$): δ = 118.68 (q, $^1J_{CF}$ = 274 Hz), 119.79 (q, $^1J_{CF}$ = 272.8 Hz), 123.32 (qq, $^2J_{CF}$ = 42.7 Hz, $^3J_{CF}$= 6.3 Hz), 129.45 (qq, $^2J_{CF}$ = 40.2 Hz, $^3J_{CF}$= 2.5 Hz).

Preparation of (*Z*)-2-bromo-1,1,1,4,4,4-hexafluorobut-2-ene (3b)

The mixture of butenes **3a**,**b** (24.29 g, 0.1 mol) was sealed in an ampoule and irradiated for several hours (completion of the reaction was monitored by ^{19}F NMR spectra), affording (*Z*)-2-bromo-1,1,1,4,4,4-hexafluorobutene (**3b**). The olefin **3b** was distilled at 52 °C, yielding 24.29 g (100%). ^{1}H NMR (400 MHz, $CDCl_3$): δ = 7.01 (q, $^{3}J_{HF}$ = 6.4 Hz, 1H); ^{19}F NMR (376.5 MHz, $CDCl_3$): δ = –61.69 (d, $^{3}J_{FH}$ = 6.4 Hz, 3F), –69.55 (s, 3F); ^{13}C NMR (125.67 MHz, $CDCl_3$): δ = 119.48 (q, $^{1}J_{CF}$ = 272.8 Hz), 120.69 (q, $^{1}J_{CF}$ = 271.5 Hz), 121.61 (qq, $^{2}J_{CF}$ = 39 Hz, $^{3}J_{CF}$= 6.3 Hz), 125.84 (qq, $^{2}J_{CF}$ = 37.7 Hz, $^{3}J_{CF}$= 5 Hz).

Synthesis of 2-chloro-3-iodo-1,1,1,4,4,4-hexafluorobutane (5)

(*E*) or (*Z*) 1,1,1,4,4,4-hexafluorobut-2-enes (**1a**,**b**) (16.4 g, 0.1 mol) and ICl (16.2 g, 0.1 mol) stirred under the influence of sunlight until the color disappears. The reaction mixture was washed with saturated aqueous solution of Na_2SO_3 to remove iodine residues, dried with $MgSO_4$ and distilled affording slightly pinkish 2-chloro-3-iodo-1,1,1,4,4,4-hexafluorobutane (**5**); b.p. 112 °C (750 mmHg), yield 27.7 g (85%). ^{1}H NMR (301.5 MHz, $CDCl_3$): δ = 4.27 (q, $^{3}J_{HF}$ = 6.3 Hz, 1H, isomer-1), 4.46 (qd, $^{3}J_{HF}$ = 6.3 Hz, $^{3}J_{HH}$ = 3.6 Hz, 1H, isomer-2), 4.79 (qd, $^{3}J_{HF}$ = 7.5 Hz, $^{3}J_{HH}$ = 3.6 Hz, 1H, isomer-2), 4.83 (q, $^{3}J_{HF}$ = 7.5 Hz, 1H, isomer-1); ^{19}F NMR (188.14 MHz, $CDCl_3$): δ = –63.73 (qd, $^{5}J_{FF}$ = 11.3 Hz, $^{3}J_{FH}$ = 7.5 Hz, 3F, isomer-2), –67.71 (d, $^{3}J_{FH}$ = 7.5 Hz, 3F, isomer-1), –69.90 (qd, $^{5}J_{FF}$ = 11.3 Hz, $^{3}J_{FH}$ = 6.3 Hz, 3F, isomer-2), –72.74 (d, $^{3}J_{FH}$ = 6.3 Hz, 3F, isomer-1); ^{13}C NMR (125.67 MHz, $CDCl_3$): δ = 16.18 (q, $^{2}J_{CF}$ = 33.9 Hz, isomer-2), 17.57 (qq, $^{2}J_{CF}$ = 31.4 Hz, $^{3}J_{CF}$ = 2.5 Hz, isomer-1), 53.98 (qq, $^{2}J_{CF}$ = 35.2 Hz, $^{3}J_{CF}$ = 2.5 Hz, isomer-1), 57.32 (qq, $^{2}J_{CF}$ = 35.2 Hz, $^{3}J_{CF}$ = 1.3, isomer-2), 121.04 (q, $^{1}J_{CF}$ = 281.5 Hz, isomer-2), 121.54 (q, $^{1}J_{CF}$ = 280.2 Hz, isomer-1), 122.06 (q, $^{1}J_{CF}$ = 279 Hz, isomer-2), 122.35 (q, $^{1}J_{CF}$ = 277.7 Hz, isomer-1); GCMS *m/z*: 325.9/327.9 $[M]^+$, 199/201 $[M\text{-}I]^+$, 161.9/163.9 $[M\text{-}ICl]^+$, 69 $[CF_3]^+$.

Dehydrohalogenation of 2-chloro-3-iodo-1,1,1,4,4,4-hexafluorobutane (5)

To butane **5** (32.6 g, 0.1 mol) suspended in 70 mL of water were added Bu_4NBr (1.61 g, 5 mmol) and a solution of KOH (7.28 g, 0.13 mol) in 35 mL of water. The reaction mixture was stirred at room temperature (completion of the reaction was monitored by ^{19}F NMR spectra). The crude products were separated from the aqueous phase, washed with saturated solution of Na_2SO_3, and dried with $MgSO_4$. The chloro- and iodo-butenes were separated by distillation at 35 °C in 52% yield (10.32 g) for **6a**,**b** and at 75 °C in 34% yield (9.86 g) for **7a**,**b**. NMR spectra of *(E)-2-chloro-1,1,1,4,4,4-hexafluorobut-2-ene* (**6a**): ^1H NMR (301.5 MHz, $CDCl_3$): δ = 6.44 (q, $^3J_{HF}$ = 7.8 Hz, 1H); ^{19}F NMR (376.5 MHz, $CDCl_3$): δ = –57.78 (qd, $^5J_{FF}$ = 11.3 Hz, $^3J_{FH}$ = 7.8 Hz, 3F), –65.53 (q, $^5J_{FF}$ = 11.3 Hz, 3F); ^{13}C NMR (150.8 MHz, $CDCl_3$): δ = 118.39 (qq, $^1J_{CF}$ = 274.5 Hz, $^4J_{CF}$ = 1.5 Hz), 119.53 (q, $^1J_{CF}$ = 272.9 Hz), 125.28 (qq, $^2J_{CF}$ = 40.7 Hz, $^3J_{CF}$ = 3 Hz), 133.69 (qq, $^2J_{CF}$ = 42.2 Hz, $^3J_{CF}$ = 6 Hz). NMR spectra of *(Z)-2-chloro-1,1,1,4,4,4-hexafluorobut-2-ene* (**6b**) fully correspond to the literature data [1, 2] GCMS *m/z* (**6a**,**b**): 198/200 $[M]^+$, 179/181 $[M\text{-}F]^+$, 163/165 $[M\text{-}Cl]^+$, 129/131 $[M\text{-}CF_3]^+$, 113/115 $[M\text{-}CF_2Cl]^+$, 69 $[CF_3]^+$. NMR spectra of *(E)-2-iodo-1,1,1,4,4,4-hexafluorobut-2-ene* (**7a**): ^1H NMR (301.5 MHz, $CDCl_3$): δ = 6.89 (q, $^3J_{HF}$ = 7.6 Hz, 1H); ^{19}F NMR (188.14 MHz, $CDCl_3$): δ = –59.01 (qd, $^5J_{FF}$ = 11.3 Hz, $^3J_{FH}$ = 7.6 Hz, 3F), –60.88 (q, $^5J_{FF}$ = 11.3 Hz, 3F); ^{13}C NMR (125.67 MHz, $CDCl_3$): δ = 96.86 (qq, $^2J_{CF}$ = 41.5 Hz, $^3J_{CF}$ = 6.3 Hz), 118.78 (q, $^1J_{CF}$ = 274 Hz), 119.53 (q, $^1J_{CF}$ = 274 Hz); 136.92 (qq, $^2J_{CF}$ = 40.2 Hz, $^3J_{CF}$ = 2.5 Hz). NMR spectra of *(Z)-2-iodo-1,1,1,4,4,4-hexafluorobut-2-ene* (**7b**): ^1H NMR (301.5 MHz, $CDCl_3$): δ = 7.18 (qq, $^3J_{HF}$ = 6.6 Hz, $^4J_{HF}$ = 1.2 Hz, 1H); ^{19}F NMR (188.14 MHz, $CDCl_3$): δ = –61.97 (d, $^3J_{FH}$ = 6.6 Hz, 3F), –67.12 (d, $^4J_{FH}$ = 1.2 Hz, 3F); ^{13}C NMR (125.67 MHz, $CDCl_3$): δ = 95.31 (qq, $^2J_{CF}$ = 37.7 Hz, $^3J_{CF}$ = 5 Hz), 119.81 (q, $^1J_{CF}$ = 274 Hz), 120.04 (q, $^1J_{CF}$ = 272.7 Hz), 132.1 (qq, $^2J_{CF}$ = 37.7, $^3J_{CF}$ = 5 Hz). GCMS *m/z (**7a, b**)*: 289.9 $[M]^+$, 270.9 $[M\text{-}F]^+$, 220.9 $[M\text{-}CF_3]^+$, 163 $[M\text{-}I]^+$, 144 $[M\text{-}F, I]^+$.

Synthesis of 2,3-bis(trifluoromethyl)-1-(4-fluorophenyl)prop-2-ene-1-ol (10)

To a solution of bromoolefin **3a** (1.21 g, 5 mmol) in Et_2O (20 mL) at −78 °C a 3M solution of *i*-PrMgCl in THF (2.92 mL) was added dropwise. The mixture was stirred for 1 hour at −78 °C (cooled in liquid nitrogen/ethanol bath) and then 4-fluorobenzaldehyde (**9**) (0.62 g, 5 mmol) was added. Continued stirring at −65 °C for 1 h and then the reaction mixture was heated to rt. The mixture was quenched by adding of 2N solution of HCl (5 mL) and the reaction product was extracted with diethyl ether (2 × 10 mL). The organic phases were combined, washed once with saturated aqueous solution of Na_2CO_3 (10 mL), with water (2 × 10 mL) and dried with $MgSO_4$. The solvent was removed in vacuum and the obtained crude product **10** was purified by column chromatography on SiO_2 (eluent hexane:diethyl ether = 9:1); yield 0.66 (46%). ^{1}H, ^{19}F and ^{13}C NMR spectra fully correspond to the published data [3].

Synthesis of 1,1,4,4,4-pentafluorobuta-1,2-diene (11)

A mixture of butenes **3a**,**b** (12.15 g, 0.05 mol) and 50 mL heptane was added to the flask. To the addition funnel was added a 2.5 M solution of commercial *n*-BuLi in hexane (24 mL). Then the *n*-BuLi solution was added dropwise at −80 °C at such a rate that the temperature was in the range from −75 to −85 °C (cooled in liquid nitrogen/ethanol bath). The slightly cloudy, yellowish solution was allowed to stir at the same temperature for an additional 40 minutes and then the reaction mixture was heated to rt. The volatile products were removed from the solution at 20 °C for 3–4 h with a slow flow of argon through the system and condensed into a trap cooled with dry ice. The product **11** was recondensed twice to separate as much as possible from solvent residues. A total of 4 g (55%) of 1,1,4,4,4-pentafluorobuta-1,2-diene (**11**) was obtained with 95% purity; b.p. 7 °C (750 mm Hg). ^{1}H NMR (400 MHz, C_6D_6): δ 6.43 (m, 1H); ^{19}F (376,5 MHz, C_6D_6): δ −64.97 (m, 3F), −97.54 (m, 2F); ^{13}C (150,8 MHz, C_6D_6): δ 108.3 (qt, ^2J = 41 Hz, ^3J =

4.5 Hz, =C(H)CF_3), 119.1 (qt, ^1J = 271 Hz, ^4J = 3 Hz, -CF_3), 155.2 (tq, ^1J = 271 Hz, ^4J = 1.5 Hz, =CF_2), 180.8 (tq, ^2J = 41 Hz, ^3J = 6 Hz, =C=CF_2). IR (neat): 2038, 1283, 1253, 1145, 866, 823 cm^{-1}.

Synthesis of (*Z*)-1,2-dibromo-1,1,4,4,4-pentafluorobut-2-ene (13)

To allene **11** (4.32 g, 0.03 mol) Br_2 (4.79 g, 0.03 mol) was added dropwise at −30 °C. The reaction mixture was allowed to stir an additional 1 h at the same temperature and then was heated to rt. The crude product was washed with saturated aqueous solution of Na_2SO_3 (20 mL) and dried with $MgSO_4$. The (*Z*)-1,2-dibromo-1,1,4,4,4-pentafluorobut-2-ene (**13**) was distilled; b.p. 104 °C (750 mm Hg), yield 8.39 g (92%). ^{1}H NMR (400 MHz, $CDCl_3$): δ = 6.93 (q, $^3J_{HF}$ = 6.8 Hz, 1H); ^{19}F NMR (376.5 MHz, $CDCl_3$): δ = –50.11 (s, 2F), –59.74 (d, $^3J_{FH}$ = 6.8 Hz, 3F); ^{13}C NMR (125.67 MHz, $CDCl_3$): δ = 114.02 (t, $^1J_{CF}$ = 305.4 Hz), 120.34 (q, $^1J_{CF}$ = 271.4 Hz), 122.25 (qt, $^2J_{CF}$ = 37.7 Hz, $^3J_{CF}$= 6.3 Hz), 128.61 (tq, $^2J_{CF}$ = 28.9 Hz, $^3J_{CF}$= 5 Hz); GCMS *m/z*: 303.8 $[M]^+$, 222.9/224.9 $[M\text{-}Br]^+$, 203.9/205.9 $[M\text{-}Br, F]^+$, 172.9/174.9 $[M\text{-}CF_2Br]^+$.

Reaction of 1,1,4,4,4-pentafluorobuta-1,2-diene (11) with ICl

To a solution of allene **11** (4.32 g, 0.03 mol) in pentane (30 mL) at −20 °C ICl (4.87 g, 0.03 mol) was added dropwise. The mixture was stirred for 1 hour at −20 °C and then overnight at room temperature until the reaction was completed (monitored by ^{19}F NMR spectroscopy). The reaction mixture was washed with saturated aqueous solution of Na_2SO_3 and dried with $MgSO_4$. The pentane was distilled off with a column, after which the mixture of (*Z*)-1-chloro-1,1,4,4,4-pentafluoro-2-iodobut-2-ene (**14a**), (*E*)-1-chloro-1,1,4,4,4-pentafluoro-2-iodobut-2-ene (**14b**) and 3-chloro-1,1,4,4,4-pentafluoro-2-iodobut-1-ene (**15**) was distilled; b.p 55–56 °C (100 mm Hg), yield 7.05 g (77%). NMR spectra of (*Z*)-1-chloro-1,1,4,4,4-pentafluoro-2-iodobut-2-ene (**14a**): ^{1}H NMR (301.5 MHz, $CDCl_3$): δ = 7.09 (q, $^3J_{HF}$ = 6.9 Hz, 1H); ^{19}F NMR (188.14 MHz, $CDCl_3$): δ = –52.79 (s, 2F), –61.47 (d, $^3J_{FH}$ = 6.9 Hz, 3F); ^{13}C NMR (125.67 MHz, $CDCl_3$): δ = 102.73 (tq, $^2J_{CF}$ = 30.2 Hz, $^3J_{CF}$= 5 Hz), 120.09 (q, $^1J_{CF}$ = 272.7 Hz), 123.49 (t, $^1J_{CF}$ = 291.6 Hz), 130.04 (qt, $^2J_{CF}$ =

37.7 Hz, $^3J_{CF}$= 6.3 Hz). NMR spectra of (*E*)-1-chloro-1,1,4,4,4-pentafluoro-2-iodobut-2-ene (**14b**): ^{1}H NMR (301.5 MHz, $CDCl_3$): δ = 6.75 (q, $^3J_{HF}$ = 8.1 Hz, 1H); ^{19}F NMR (188.14 MHz, $CDCl_3$): δ = –49.33 (q, $^5J_{FF}$ = 15 Hz, 2F), –59.19 (td, $^5J_{FF}$ = 15 Hz,$^3J_{FH}$ = 8.1 Hz, 3F); ^{13}C NMR (125.67 MHz, $CDCl_3$): δ = 103.82 (tq, $^2J_{CF}$ = 33.9 Hz, $^3J_{CF}$= 6.3 Hz), 119.59 (q, $^1J_{CF}$ = 275.2 Hz), 121.64 (t, $^1J_{CF}$ = 291.6 Hz), 133.59 (qt, $^2J_{CF}$ = 39 Hz, $^3J_{CF}$= 2.5 Hz). NMR spectra of 3-chloro-1,1,4,4,4-pentafluoro-2-iodobut-1-ene (**15**): ^{1}H NMR (301.5 MHz, $CDCl_3$): δ = 4.89 (qd, $^3J_{HF}$ = 6 Hz, $^4J_{HF}$ = 1.8 Hz, 1H); ^{19}F NMR (188.14 MHz, $CDCl_3$): δ = –68.52 (dm, $^2J_{FF}$ = 13.2 Hz, 1F), –72.39 (dm, $^2J_{FF}$ = 13.2 Hz, 1F), –73.65 (dm, $^3J_{FH}$ = 6 Hz, 3F); ^{13}C NMR (125.67 MHz, $CDCl_3$): δ = 42.68 (dd, $^2J_{CF}$ = 32.7 Hz, $^2J_{CF}$ = 22.6 Hz), 53.89 (q, $^2J_{CF}$ = 36.4 Hz), 121.56 (qt, $^1J_{CF}$ = 281.5 Hz, $^4J_{CF}$ = 2.5 Hz), 154.8 (dd, $^1J_{CF}$ = 301.4 Hz, $^1J_{CF}$= 289 Hz); GCMS *m/z*: 305.9/307.9 $[M]^+$, 270.9 $[M\text{-}Cl]^+$, 236.9/238.9 $[M\text{-}CF_3]^+$, 220.8 $[M\text{-}CF_2Cl]^+$, 179/181 $[M\text{-}I]^+$, 159.9/161.9 $[M\text{-}I, F]^+$.

Synthesis of 2,3-dibromo-2-chloro-1,1,1,4,4,4-hexafluorobutane (16)

The mixture of (*E*)- and (*Z*)-2-chloro-1,1,1,4,4,4-hexafluorobut-2-enes (**6a**,**b**) (9.92 g, 0.05 mol) and bromine (8 g, 0.05 mol) stirred under the influence of sunlight until the color disappears. The reaction mixture was washed with saturated aqueous solution of Na_2SO_3 to remove bromine residues, dried with $MgSO_4$ and distilled affording pure 2,3-dibromo-2-chloro-1,1,1,4,4,4-hexafluorobutane (**16**); b.p. 46 °C (20 mmHg), yield 15 g (84%). ^{1}H NMR (301.5 MHz, $CDCl_3$): δ = 4.89 (q, $^3J_{HF}$ = 6 Hz, 1H); ^{19}F NMR (188.14 MHz, $CDCl_3$): δ = –63.74 (qd, $^5J_{FF}$ = 7.5 Hz, $^3J_{FH}$ = 6 Hz, 3F, isomer-1), –63.86 (qd, $^5J_{FF}$ = 7.5 Hz, $^3J_{FH}$ = 6 Hz, 3F, isomer-2), –72.03 (q, $^5J_{FF}$ = 7.5 Hz, 3F, isomer-1), –72.35 (q, $^5J_{FF}$ = 7.5 Hz, 3F, isomer-2); ^{13}C NMR (125.67 MHz, $CDCl_3$): δ = 50.86 (q, $^2J_{CF}$ = 33.9 Hz, isomer-1), 51.12 (q, $^2J_{CF}$ = 33.9 Hz, isomer-2), 68.74 (q, $^2J_{CF}$ = 35.2 Hz, isomer-1), 69.21 (q, $^2J_{CF}$ = 35.2 Hz, isomer-2), 120.45 (q, $^1J_{CF}$ = 284 Hz), 120.87 (q, $^1J_{CF}$ = 280.2 Hz); GCMS *m/z*: 357.8 $[M]^+$, 276.9/278.9 $[M\text{-}Br]^+$, 188.9/190.9 $[M\text{-}CF_3, Br, F]^+$, 69 $[CF_3]^+$.

Dehydrobromination of 2,3-dibromo-2-chloro-1,1,1,4,4,4-hexafluorobutane (16)

To a solution of butane **16** (8.96 g, 0.025 mol) in pentane (30 mL) DBU (4.57 g, 0.03 mol) was added dropwise at rt. The reaction mixture was stirred at room temperature (completion of the reaction was monitored by ^{19}F NMR spectra). Pentane and volatile products were condensed into a trap cooled with liquid nitrogen under reduced pressure. The pentane was distilled off with a column, after which the mixture of (*Z*)- and (*E*)-2-bromo-3-chloro-1,1,1,4,4,4-hexafluorobut-2-enes (**17a**,**b**) was distilled at 78-79 °C, yield 5.41 g (78%). NMR spectra of (*Z*)-2-bromo-3-chloro-1,1,1,4,4,4-hexafluorobut-2-ene (**17a**): ^{19}F NMR (188.14 MHz, $CDCl_3$): δ = –55.77 (q, $^5J_{FF}$ = 13.2 Hz, 3F), –58.06 (q, $^5J_{FF}$ = 13.2 Hz, 3F); ^{13}C NMR (125.67 MHz, $CDCl_3$): δ = 118.19 (q, $^1J_{CF}$ = 276.5 Hz), 118.65 (q, $^1J_{CF}$ = 275.2 Hz), 122.14 (qq, $^2J_{CF}$ = 41.5 Hz, $^3J_{CF}$= 5 Hz), 133.04 (qq, $^2J_{CF}$ = 42.7 Hz, $^3J_{CF}$= 5 Hz). NMR spectra of (*E*)-2-bromo-3-chloro-1,1,1,4,4,4-hexafluorobut-2-ene (**17b**): ^{19}F NMR (188.14 MHz, $CDCl_3$): δ = –59.28 (s, 3F), –61.35 (s, 3F); ^{13}C NMR (125.67 MHz, $CDCl_3$): δ = 114.87 (qq, $^2J_{CF}$ = 39 Hz, $^3J_{CF}$= 2.5 Hz), 118.85 (q, $^1J_{CF}$ = 276.5 Hz), 118.99 (q, $^1J_{CF}$ = 276.5 Hz), 127.52 (qq, $^2J_{CF}$ = 39 Hz, $^3J_{CF}$= 5 Hz). GCMS *m/z*: 275.9/277.9/279.9 $[M]^+$, 256.8/258.8 $[M-F]^+$, 206.8/208.8/210.8 $[M-CF_3]^+$, 196.9/198.9 $[M-Br]^+$, 146.9/148.9 $[M-CF_2Br]^+$, 69 $[CF_3]^+$.

2. Copies of NMR and IR spectra of all new compounds

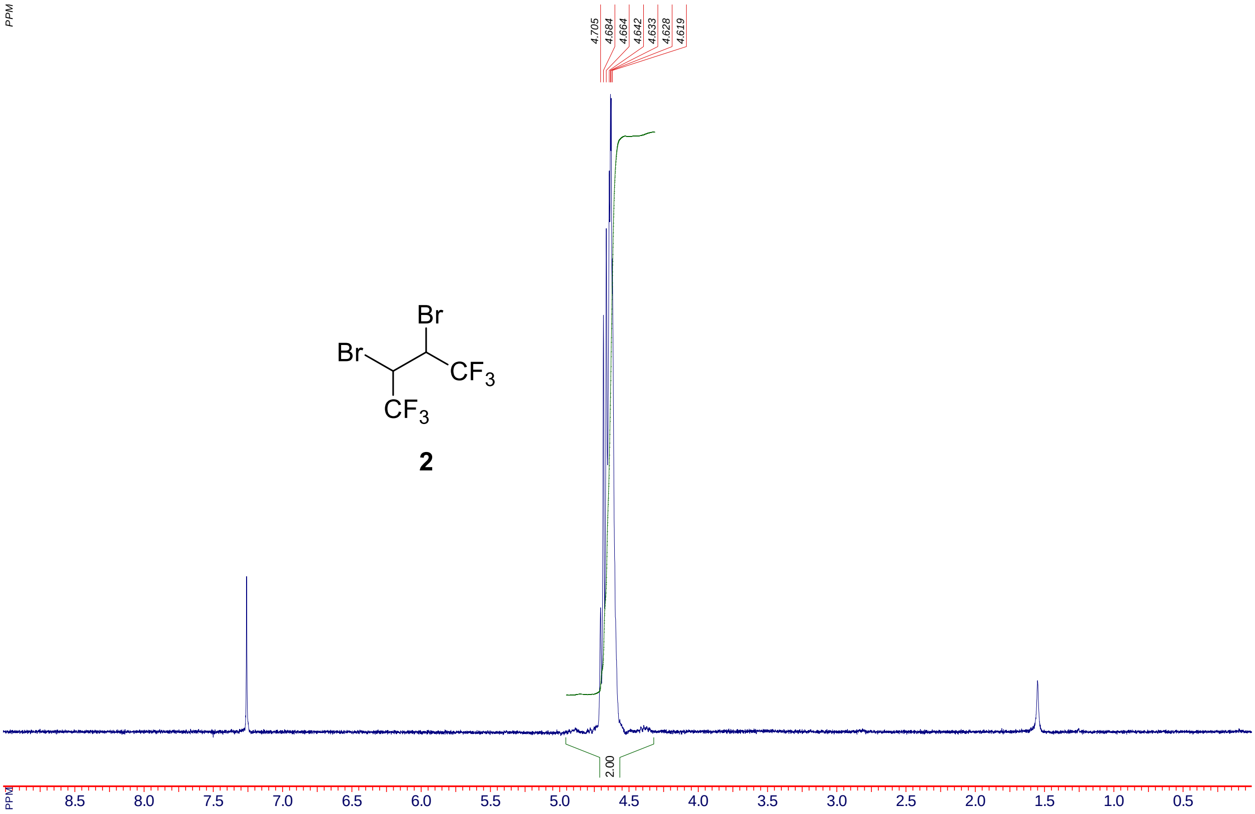

Figure S1. ^{1}H NMR of **2** ($CDCl_3$, 301.5 MHz).

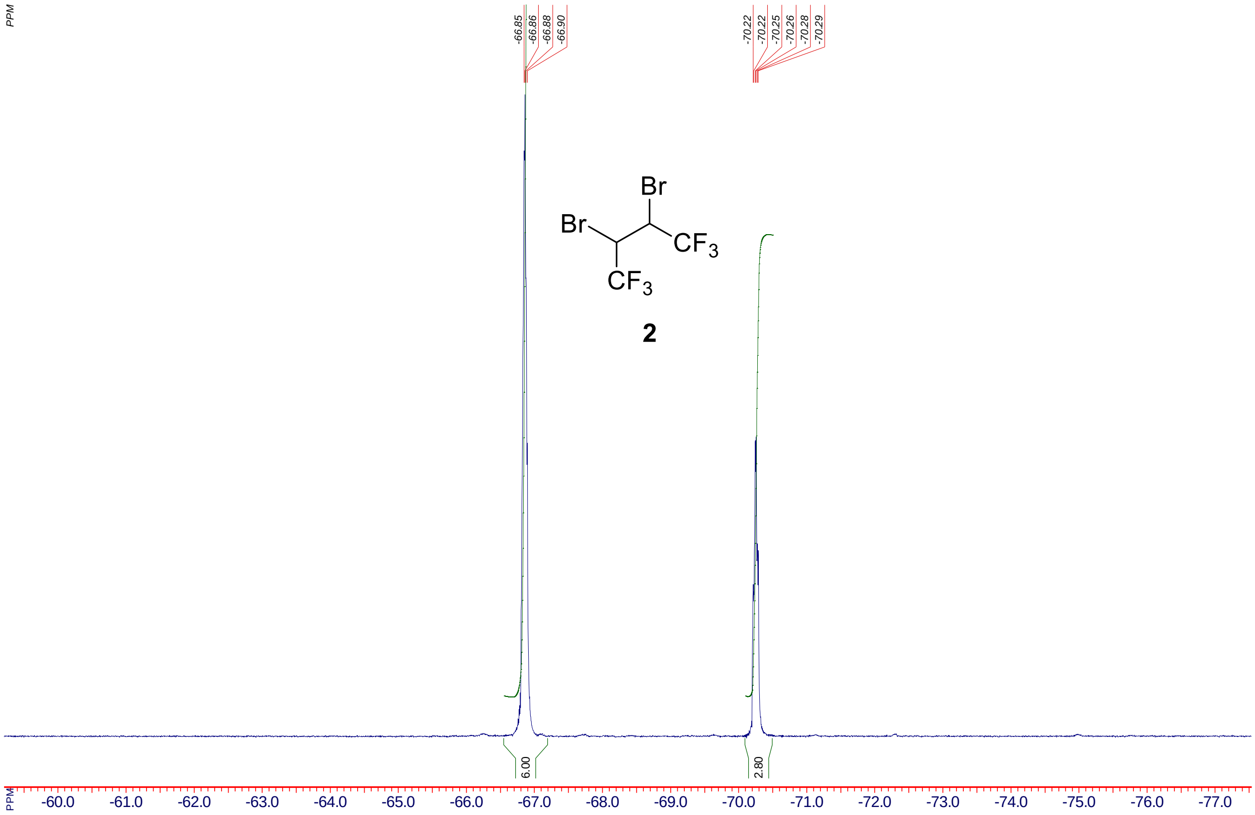

Figure S2. ^{19}F NMR of **2** ($CDCl_3$, 188.14 MHz).

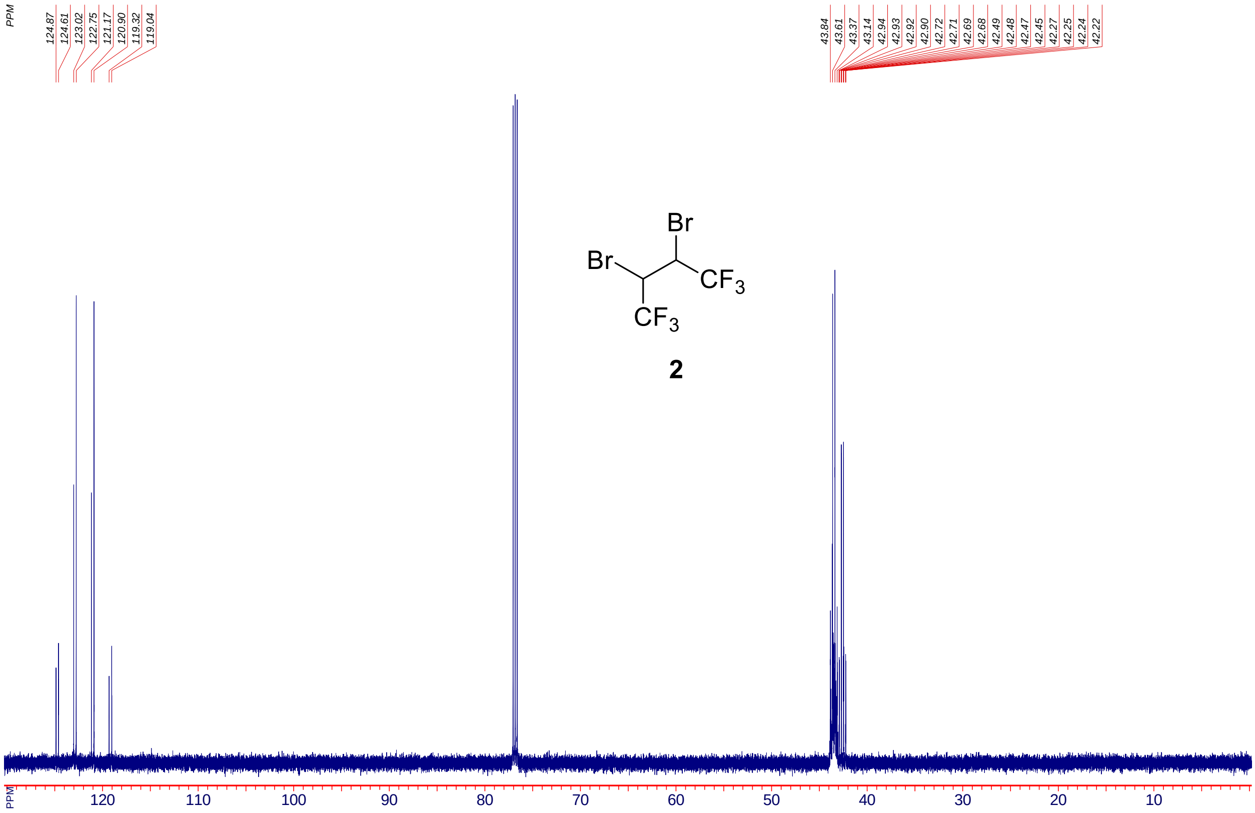

Figure S3. ^{13}C NMR of **2** ($CDCl_3$, 150.8 MHz).

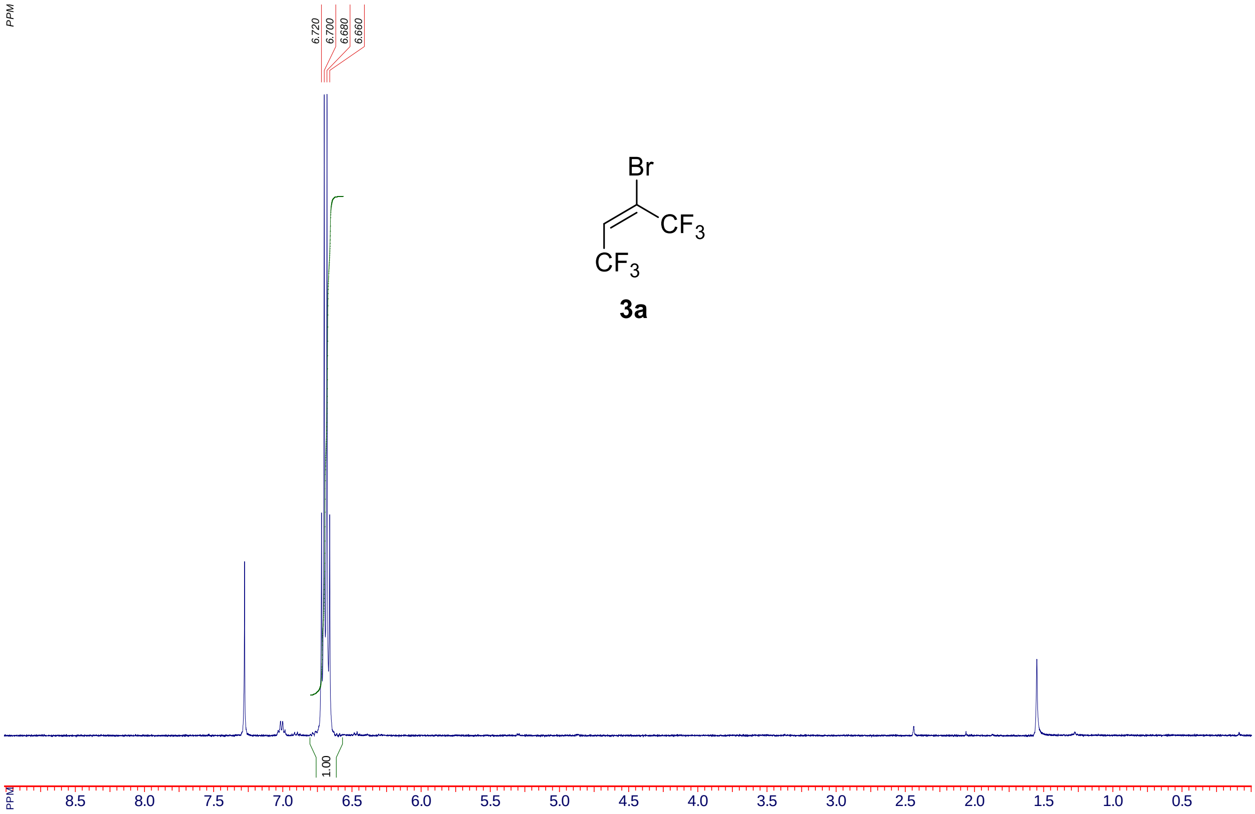

Figure S4. ^{1}H NMR of **3a** ($CDCl_3$, 400 MHz).

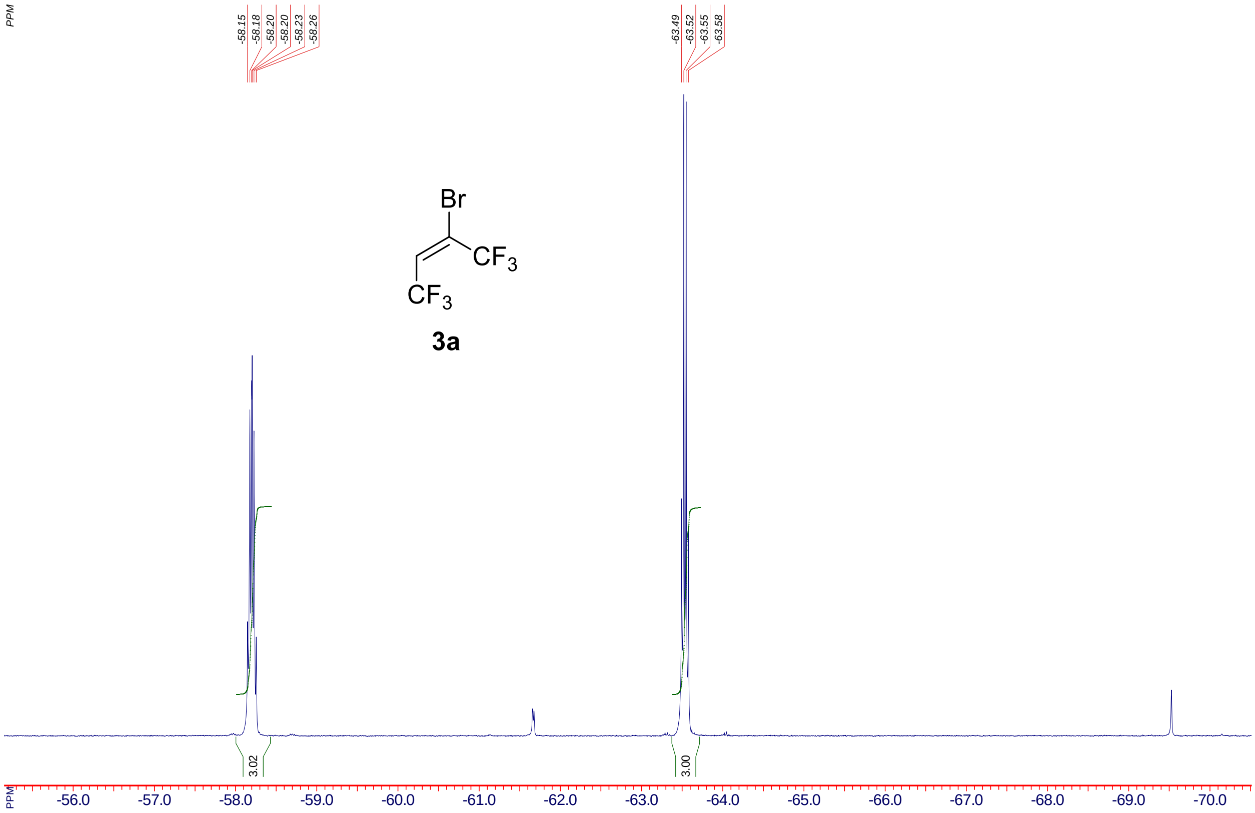

Figure S5. ^{19}F NMR of **3a** ($CDCl_3$, 376.5 MHz).

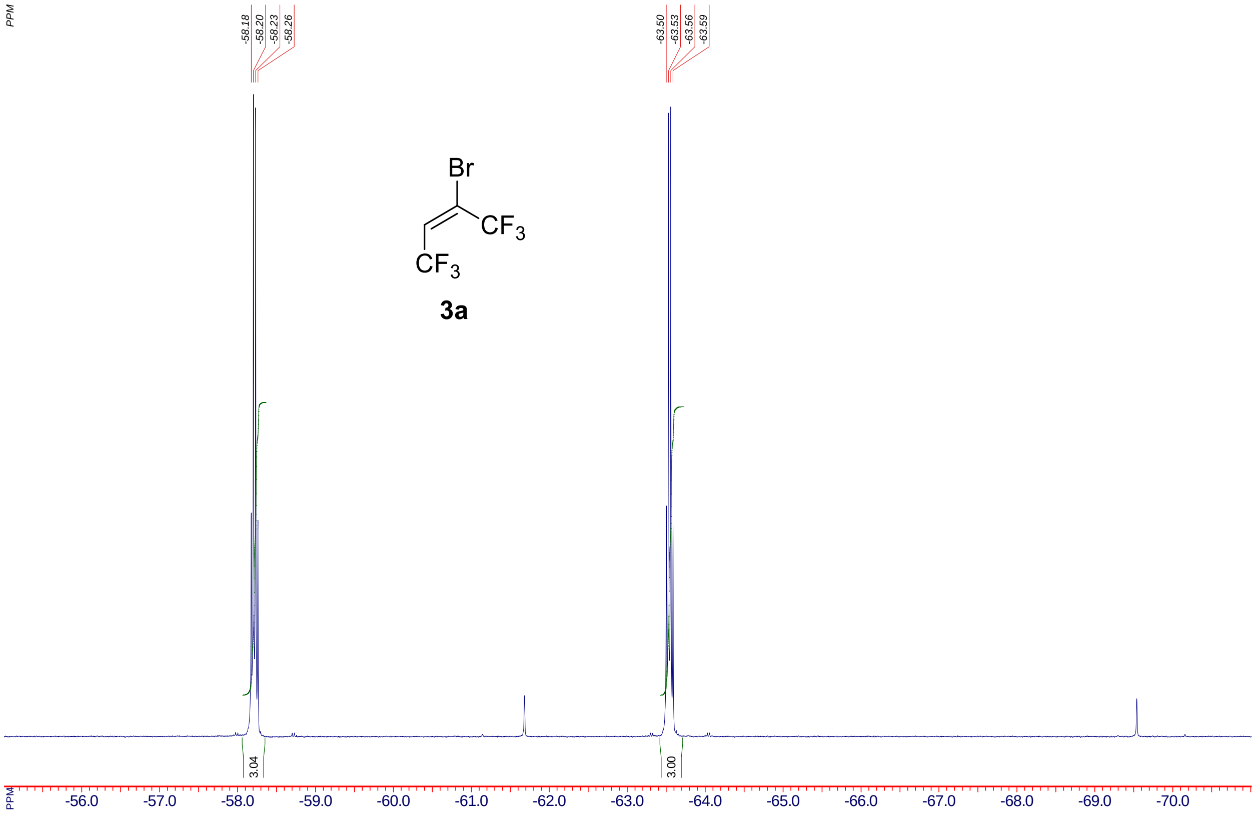

Figure S6. $^{19}F\{H\}$ NMR of **3a** ($CDCl_3$, 376.5 MHz).

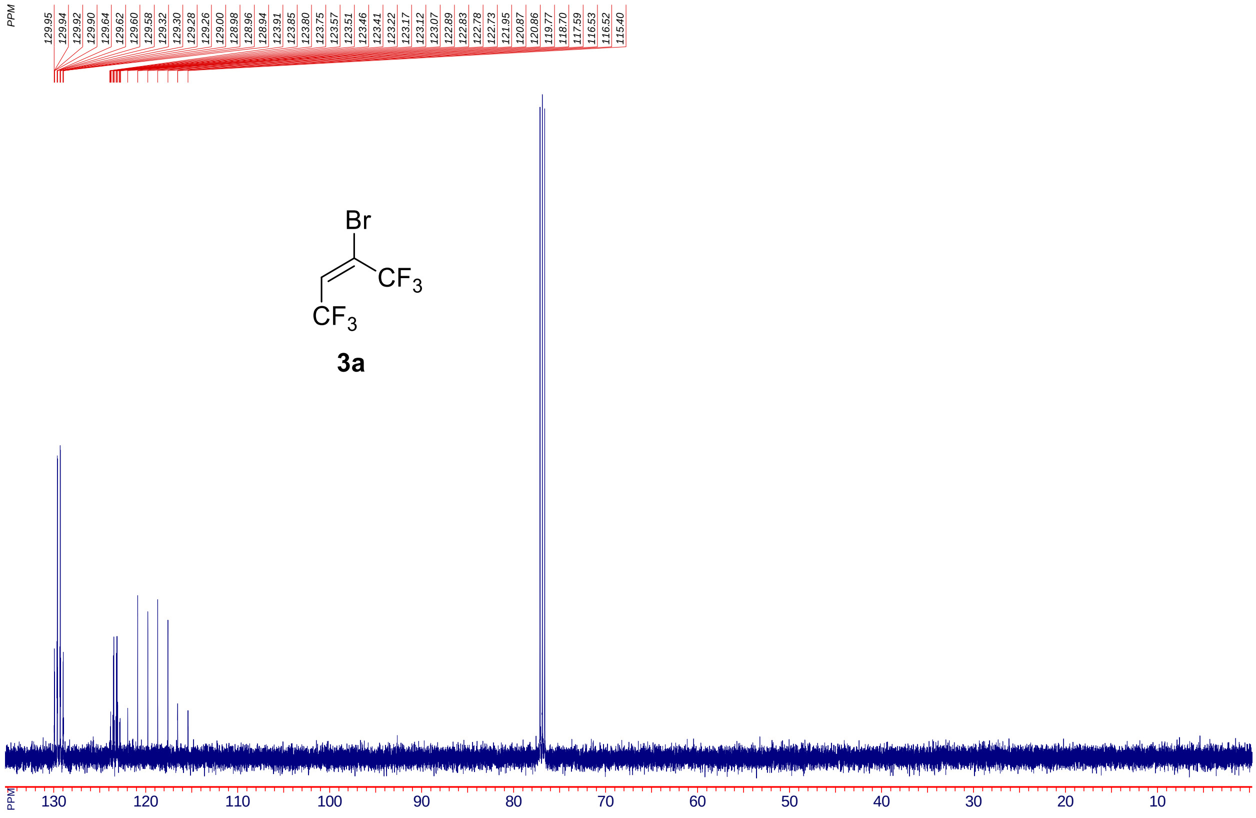

Figure S7. ^{13}C NMR of **3a** ($CDCl_3$, 125.67 MHz).

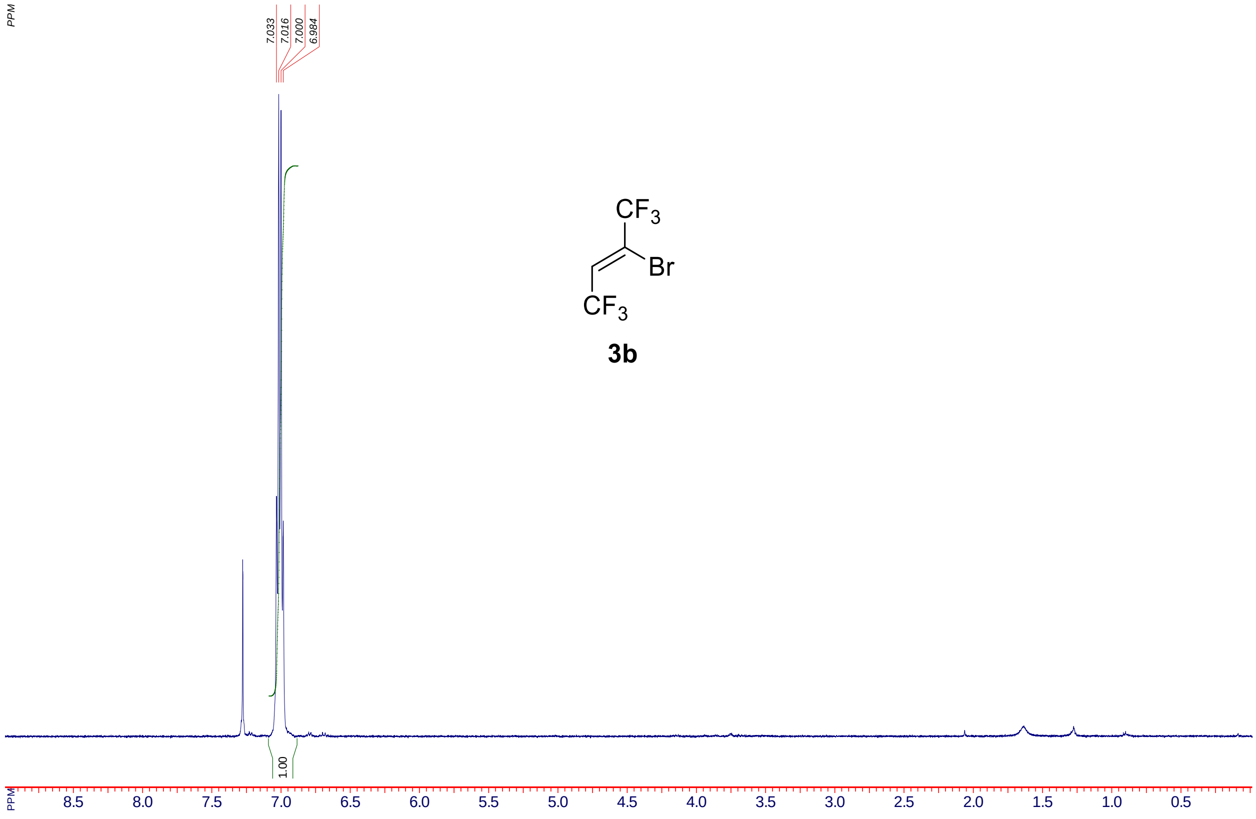

Figure S8. ^{1}H NMR of **3b** ($CDCl_3$, 400 MHz).

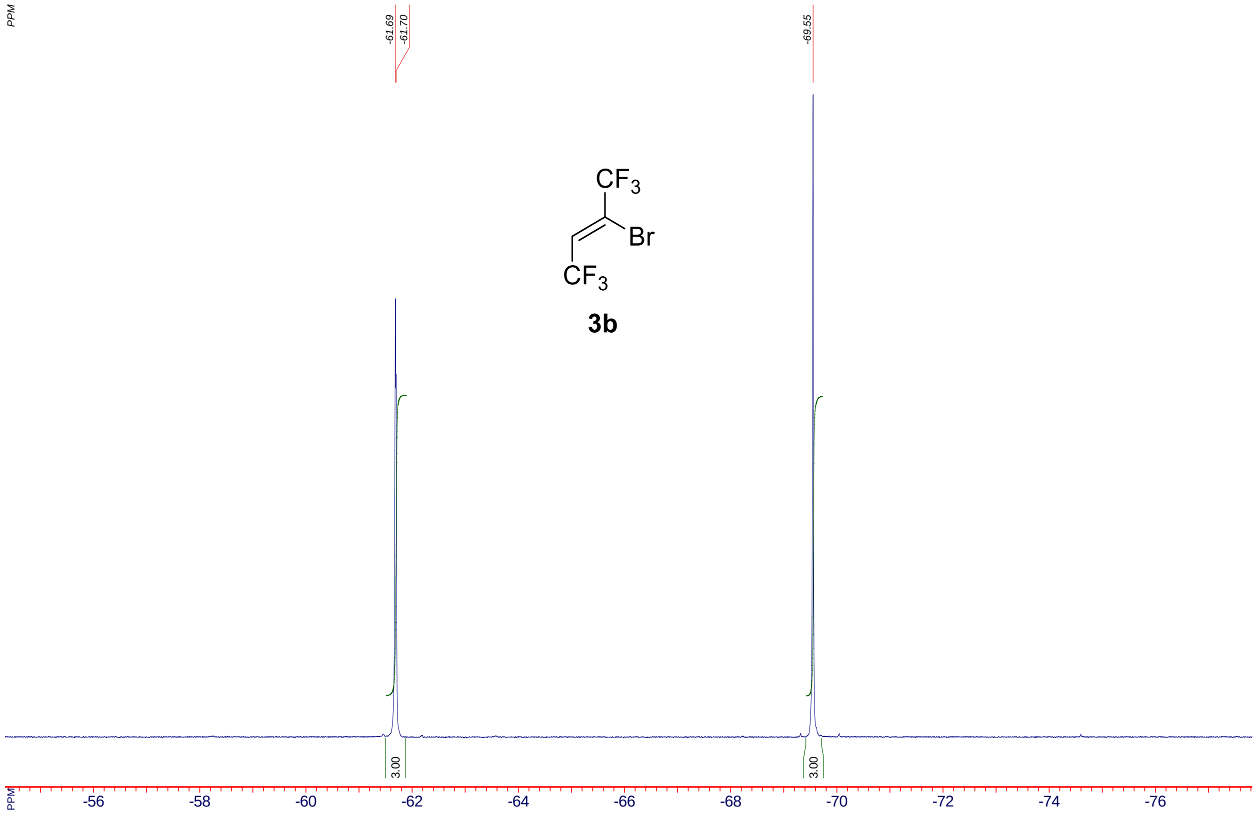

Figure S9. ^{19}F NMR of **3b** ($CDCl_3$, 376.5 MHz).

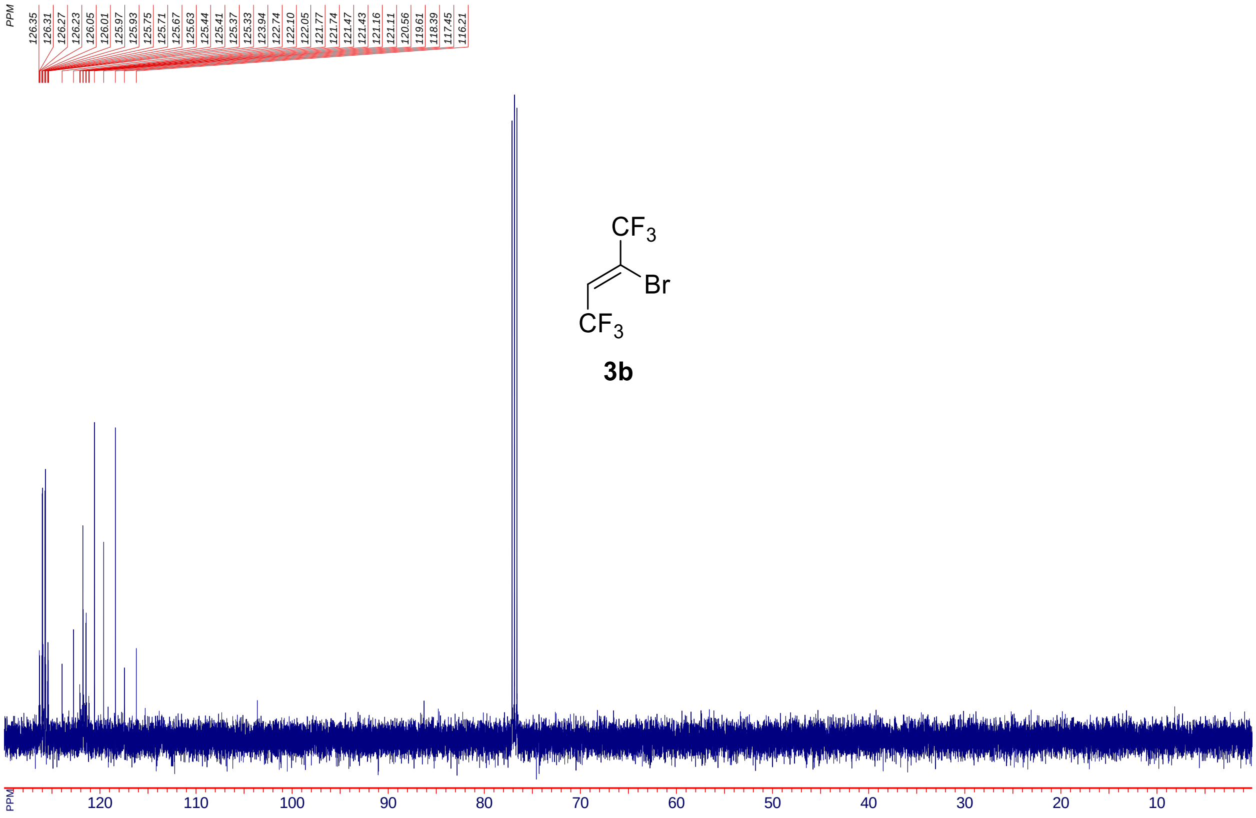

Figure S10. ^{13}C NMR of **3b** ($CDCl_3$, 125.67 MHz).

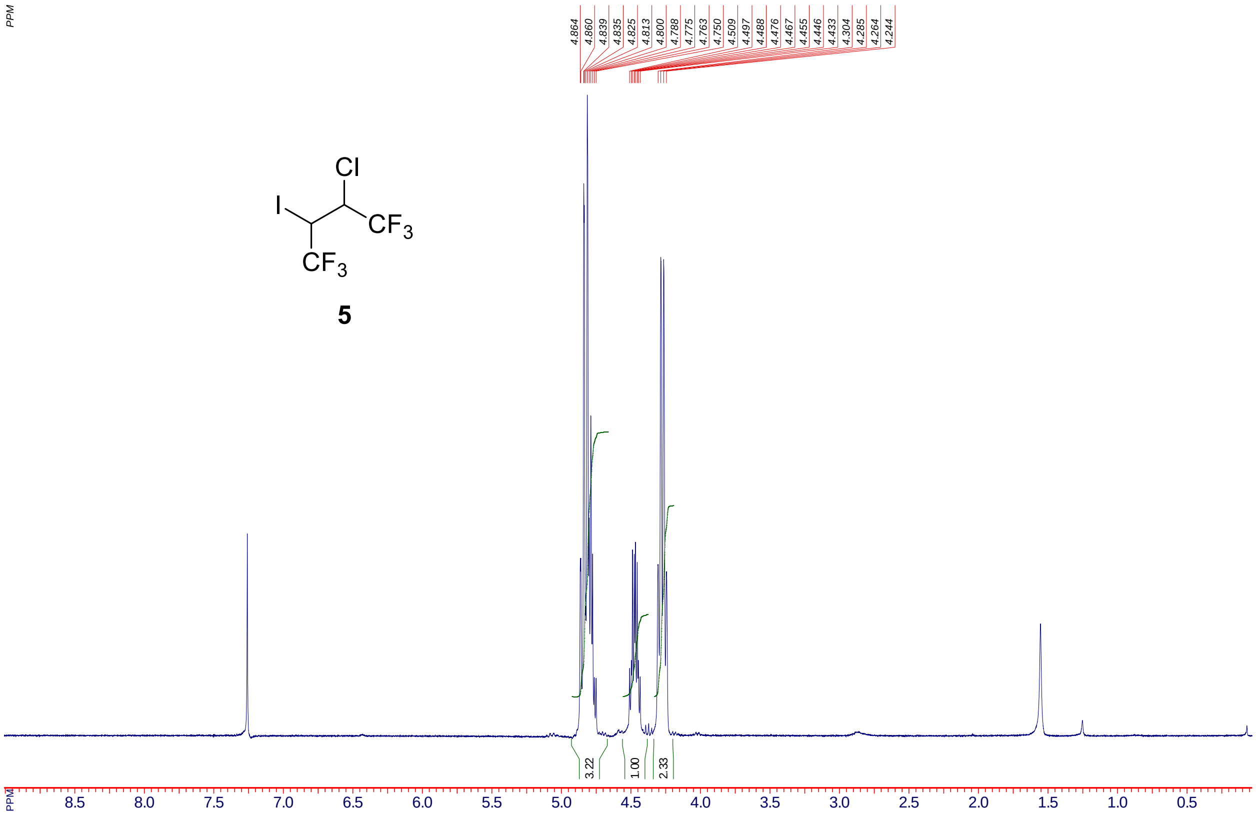

Figure S11. ^{1}H NMR of **5** ($CDCl_3$, 301.5 MHz).

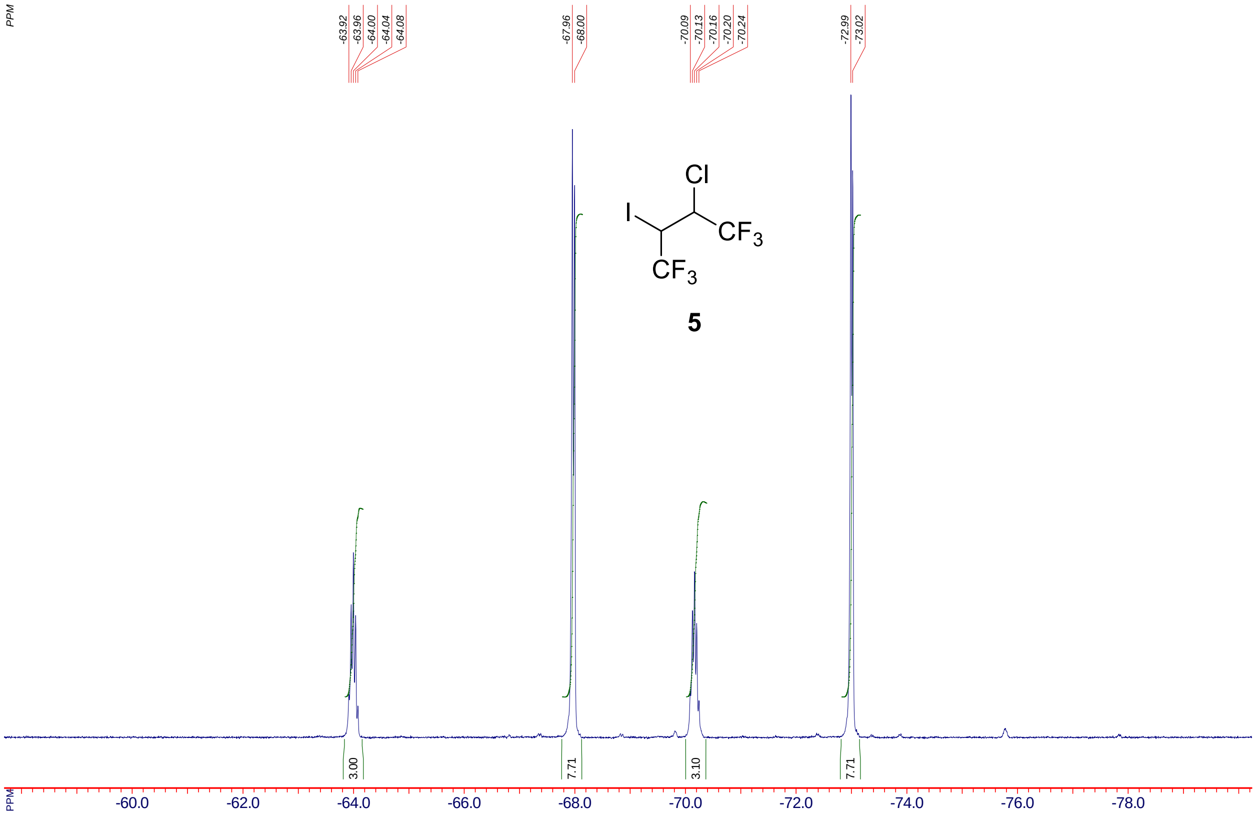

Figure S12. ^{19}F NMR of **5** ($CDCl_3$, 376.5 MHz).

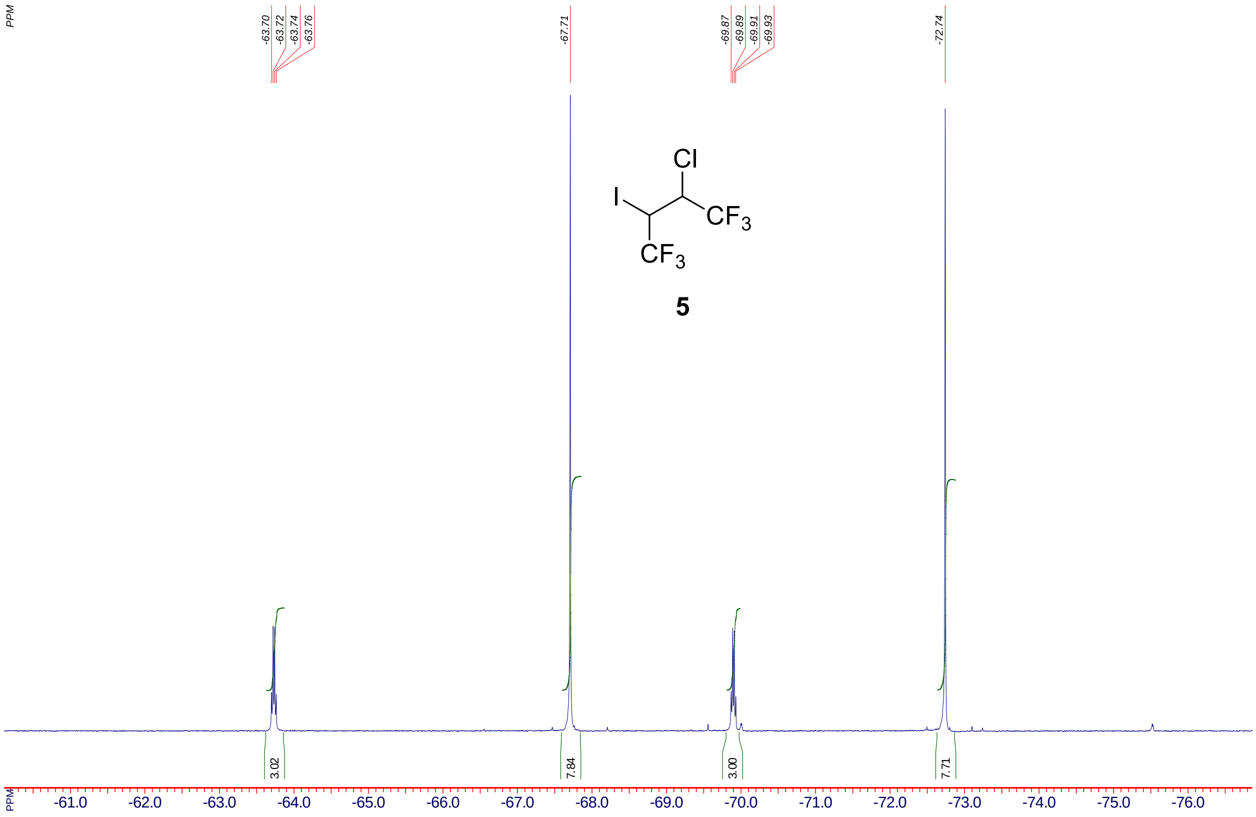

Figure S13. $^{19}F\{H\}$ NMR of **5** ($CDCl_3$, 376.5 MHz).

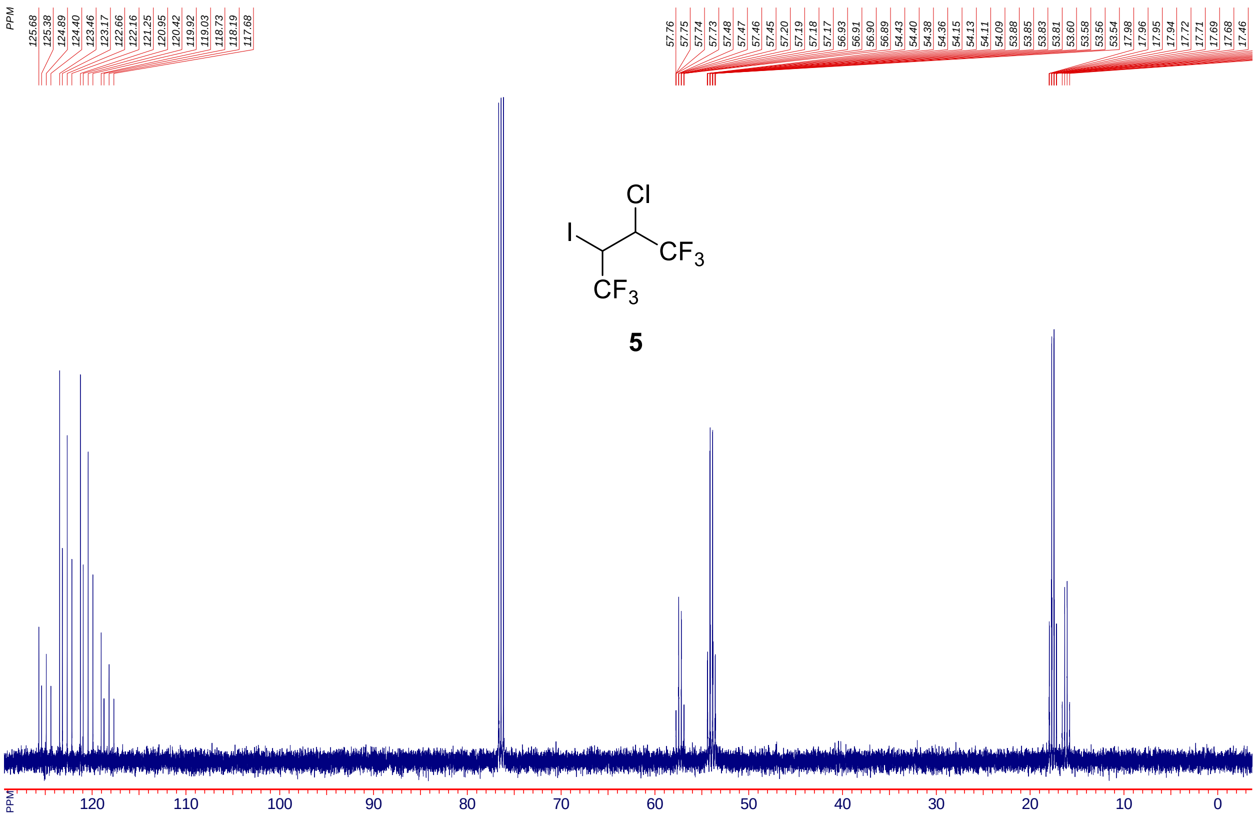

Figure S14. ^{13}C NMR of **5** ($CDCl_3$, 125.67 MHz).

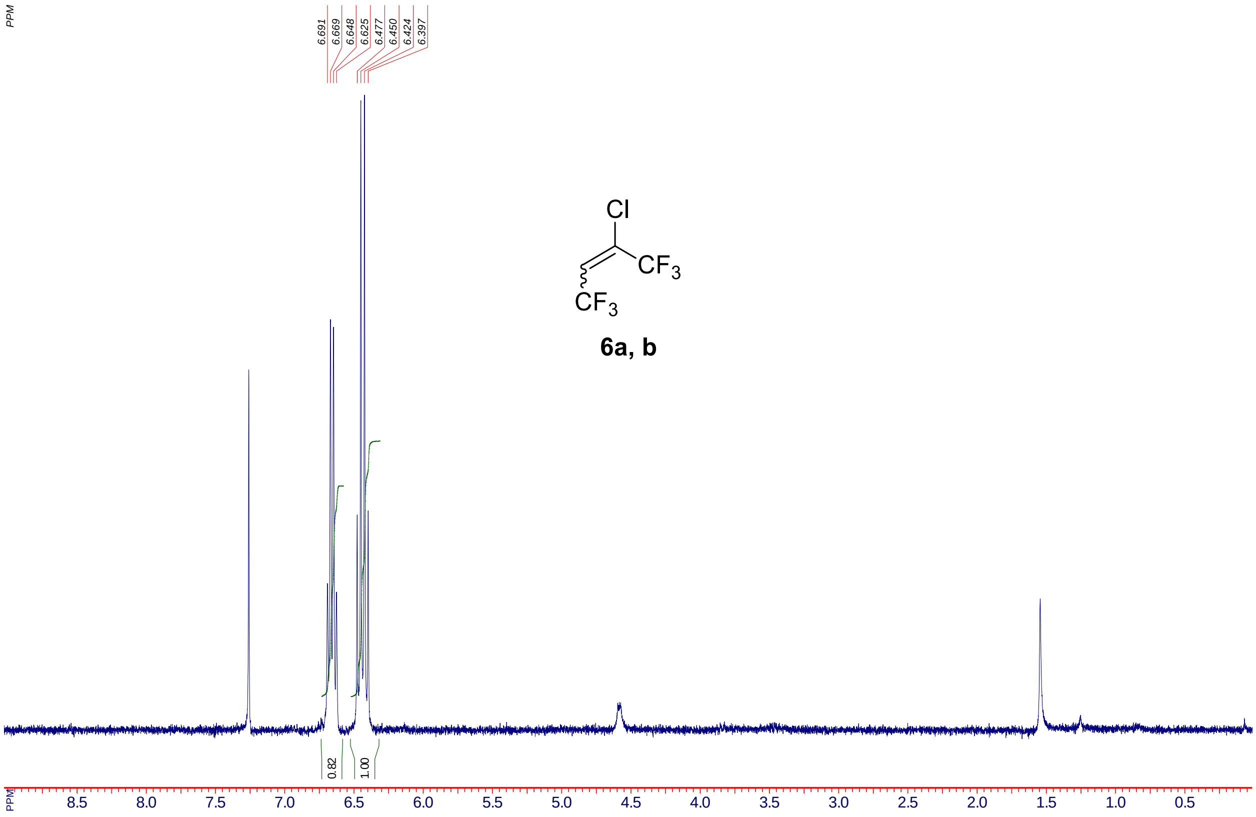

Figure S15. ^{1}H NMR of **6a, b** ($CDCl_3$, 301.5 MHz).

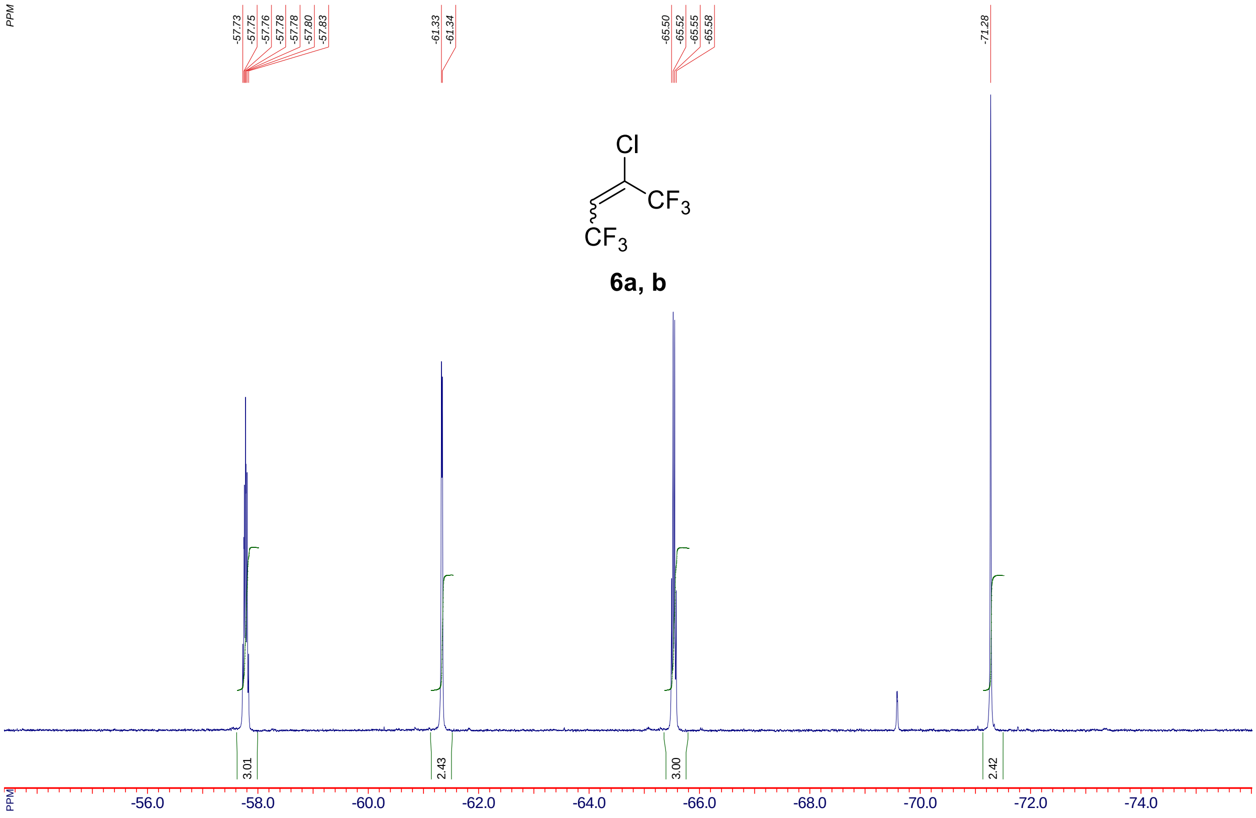

Figure S16. ^{19}F NMR of **6a, b** ($CDCl_3$, 376.5 MHz).

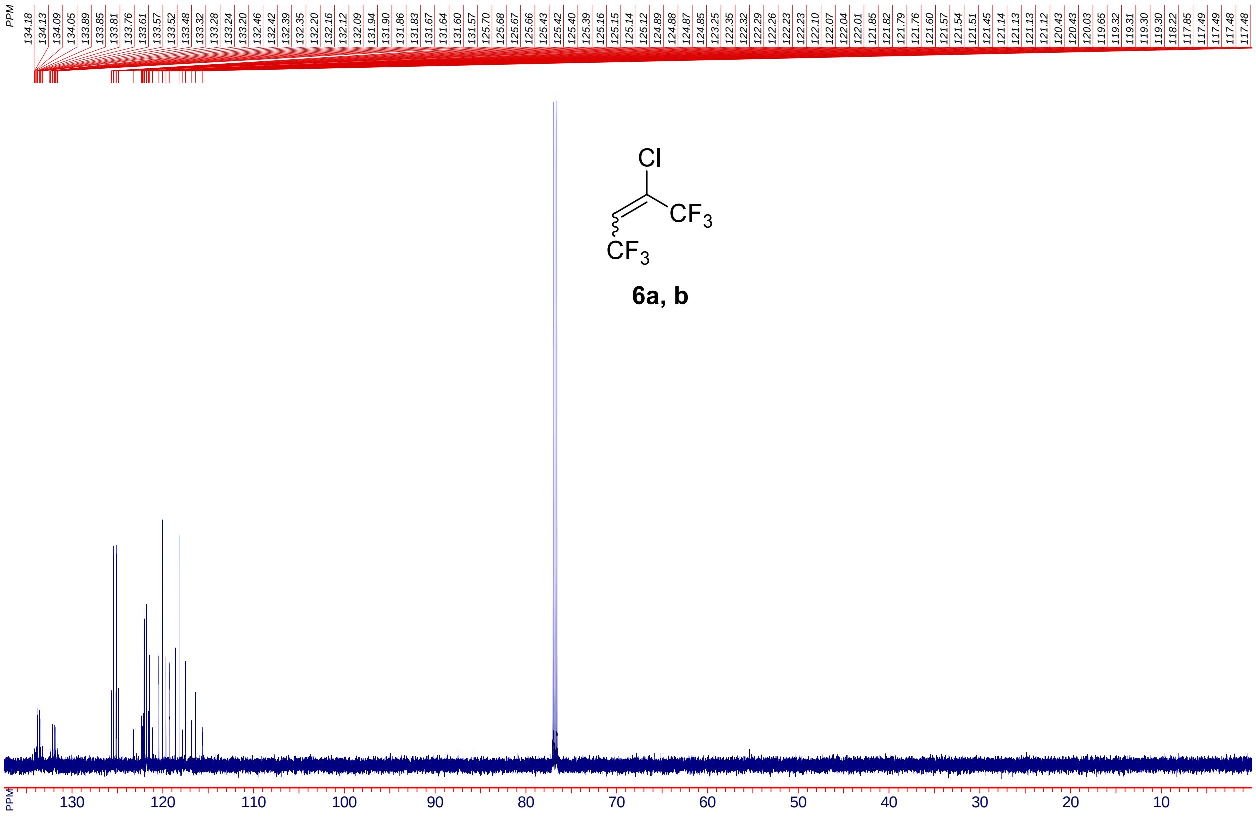

Figure S17. ^{13}C NMR of **6a, b** ($CDCl_3$, 150.8 MHz).

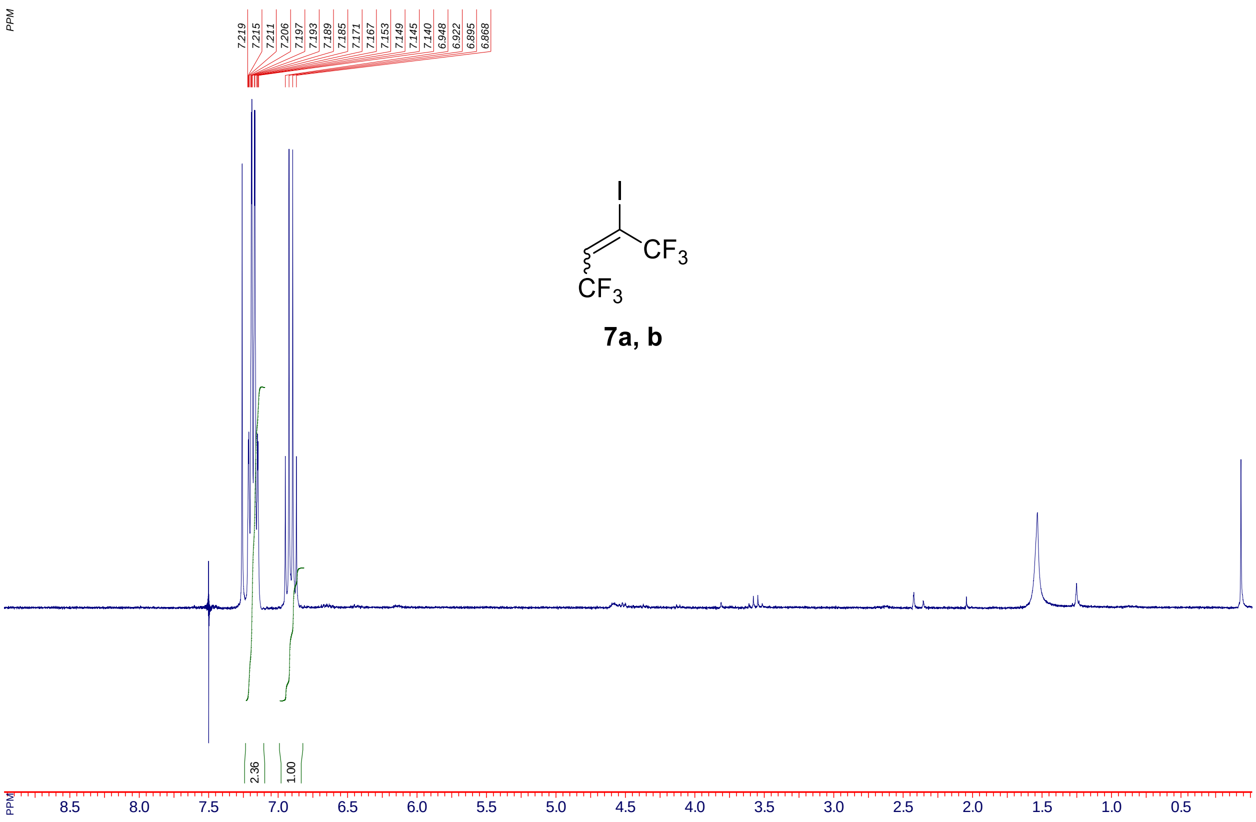

Figure S18. ^{1}H NMR of **7a, b** ($CDCl_3$, 301.5 MHz).

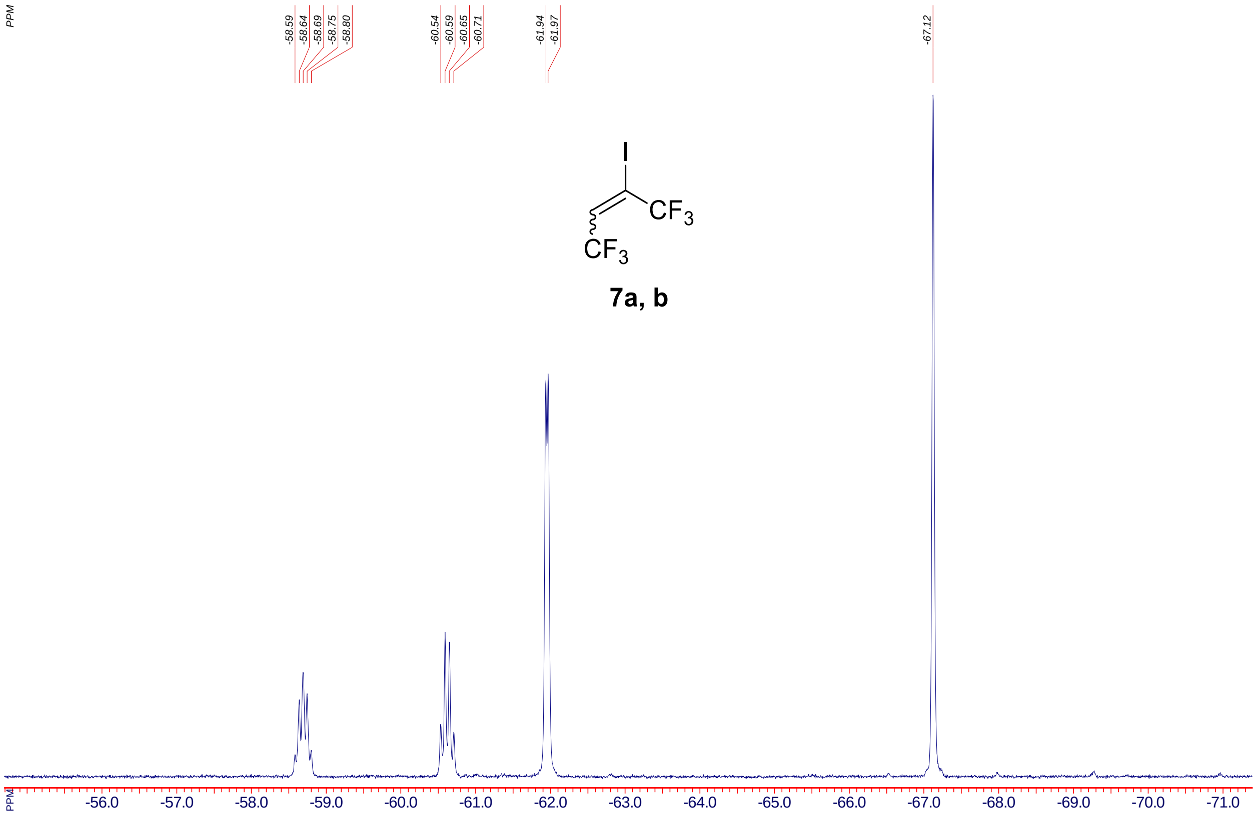

Figure S19. ^{19}F NMR of **7a, b** ($CDCl_3$, 188.14 MHz).

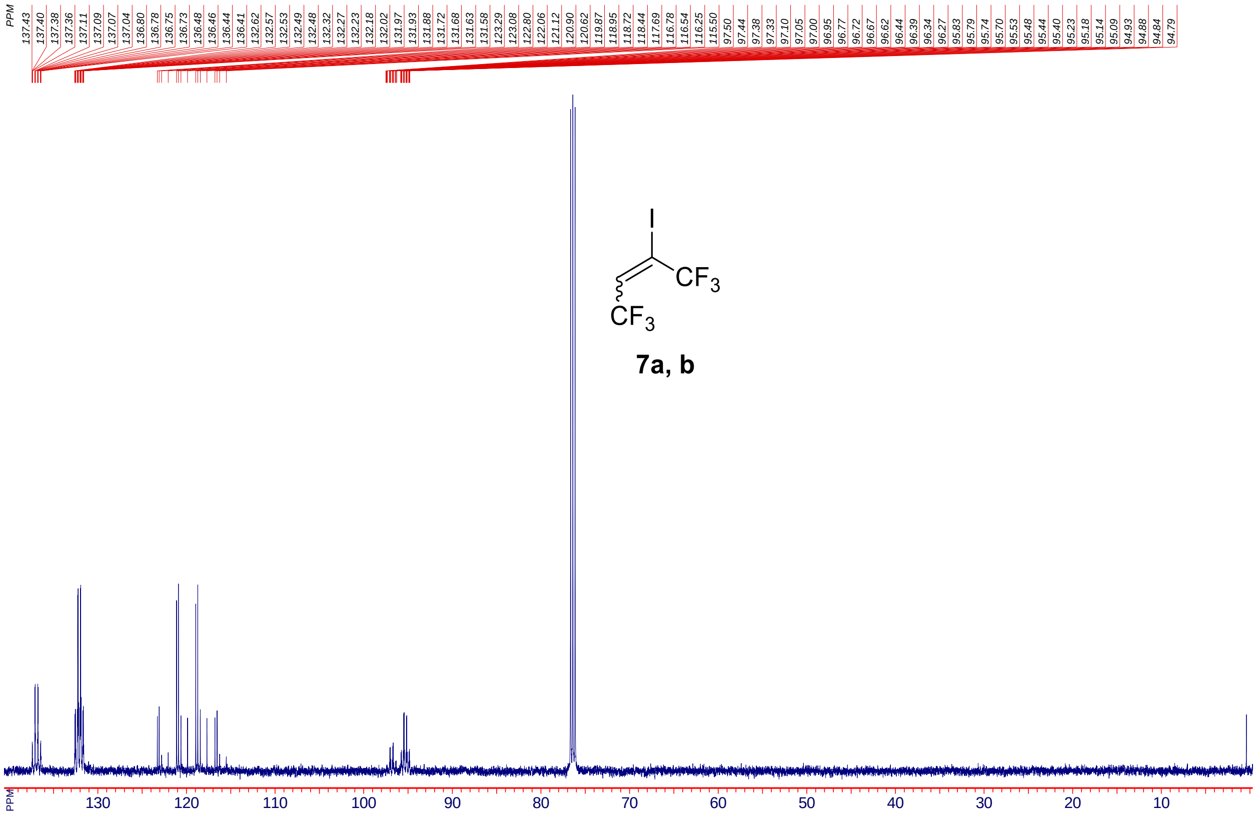

Figure S20. ^{13}C NMR of **7a, b** ($CDCl_3$, 125.67 MHz).

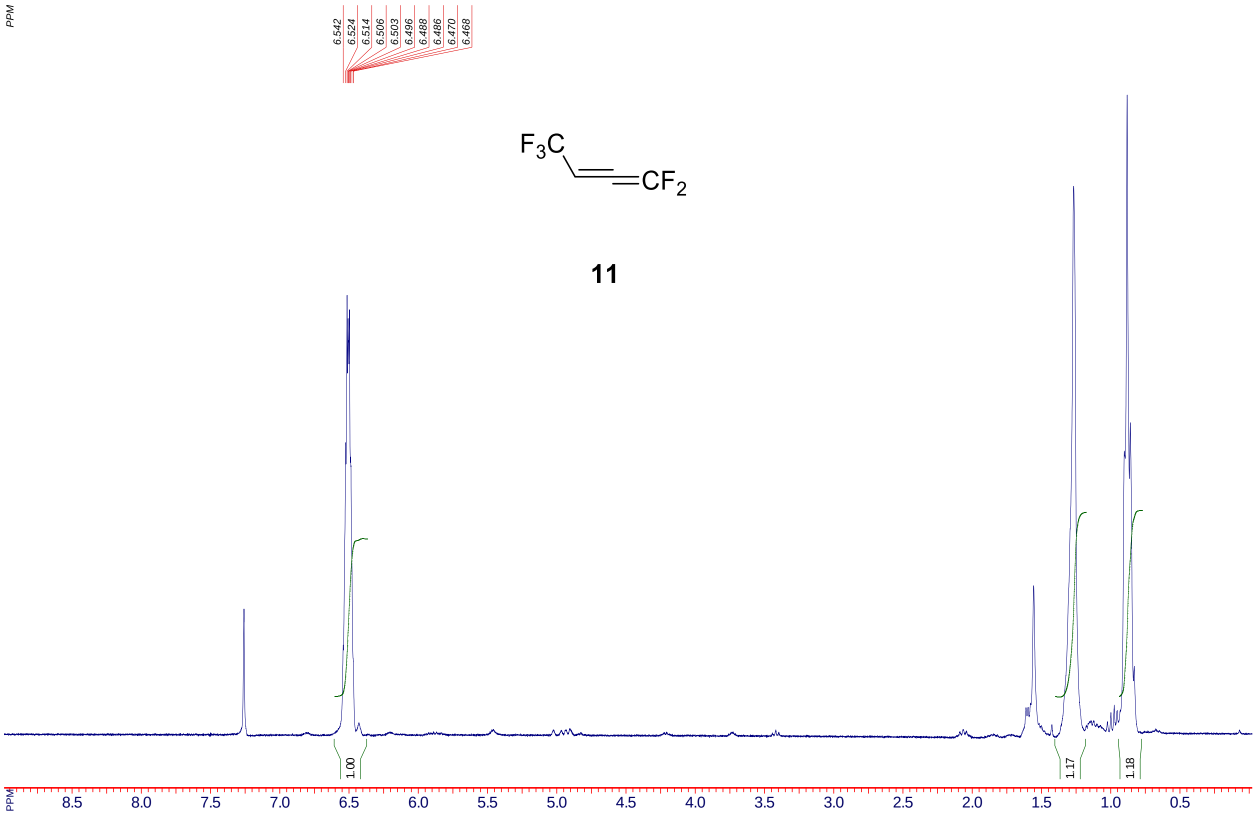

Figure S21. ^{1}H NMR of **11** ($CDCl_3$, 301.5 MHz).

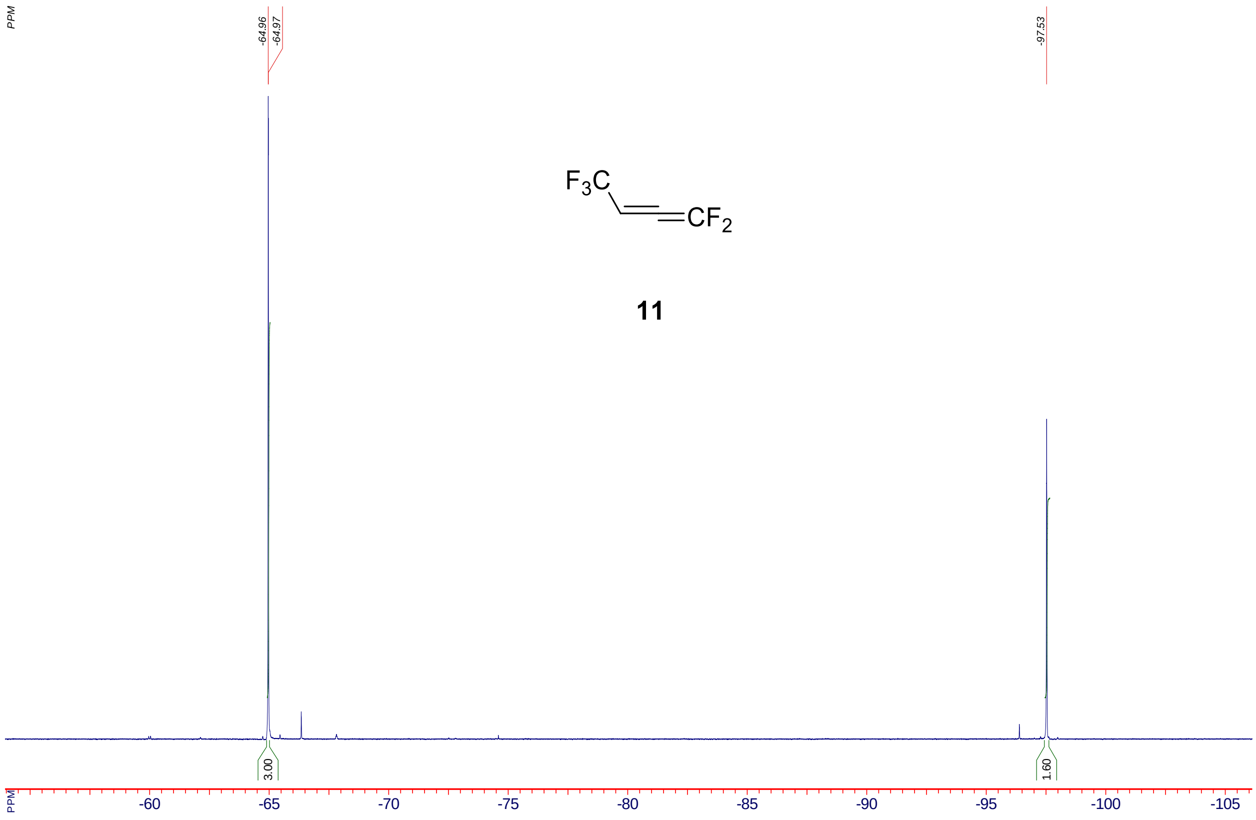

Figure S22. ^{19}F NMR of **11** ($CDCl_3$, 376.5 MHz).

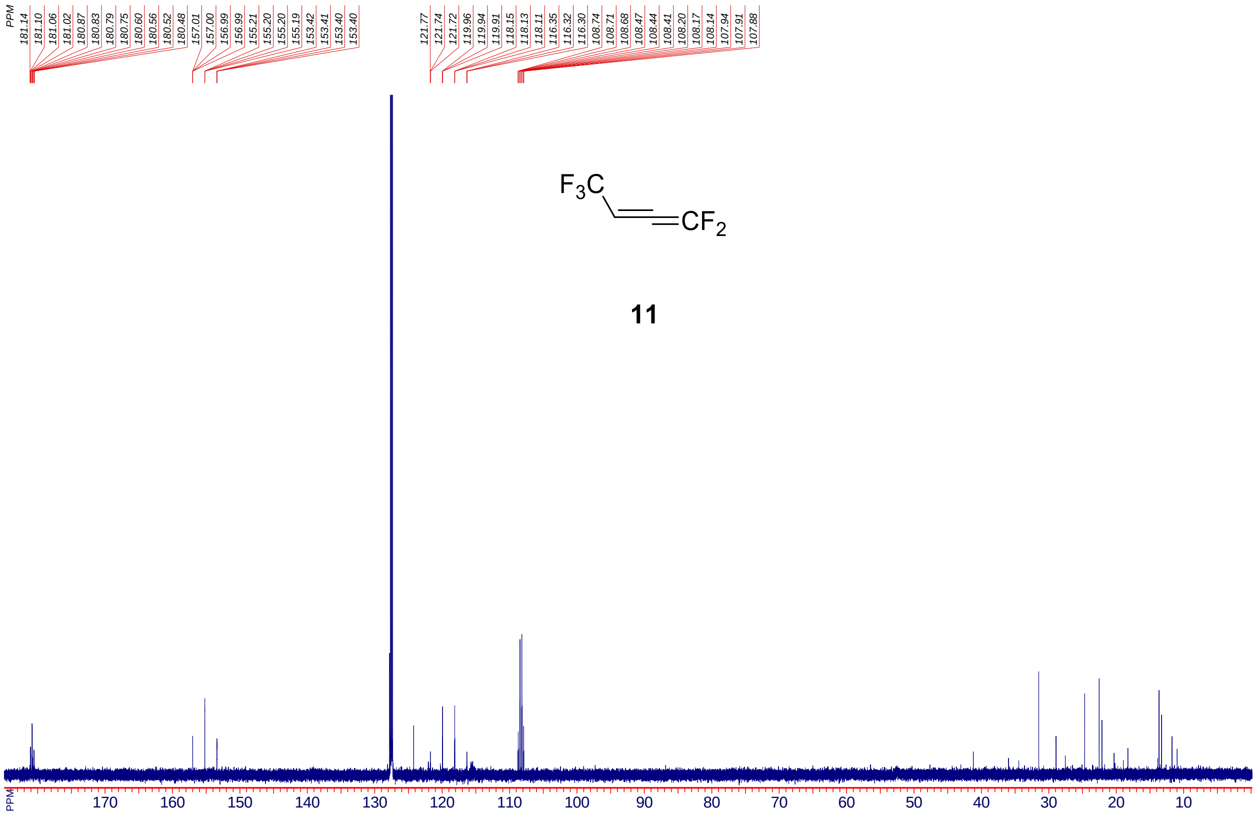

Figure S23. ^{13}C NMR of **11** ($CDCl_3$, 150.8 MHz).

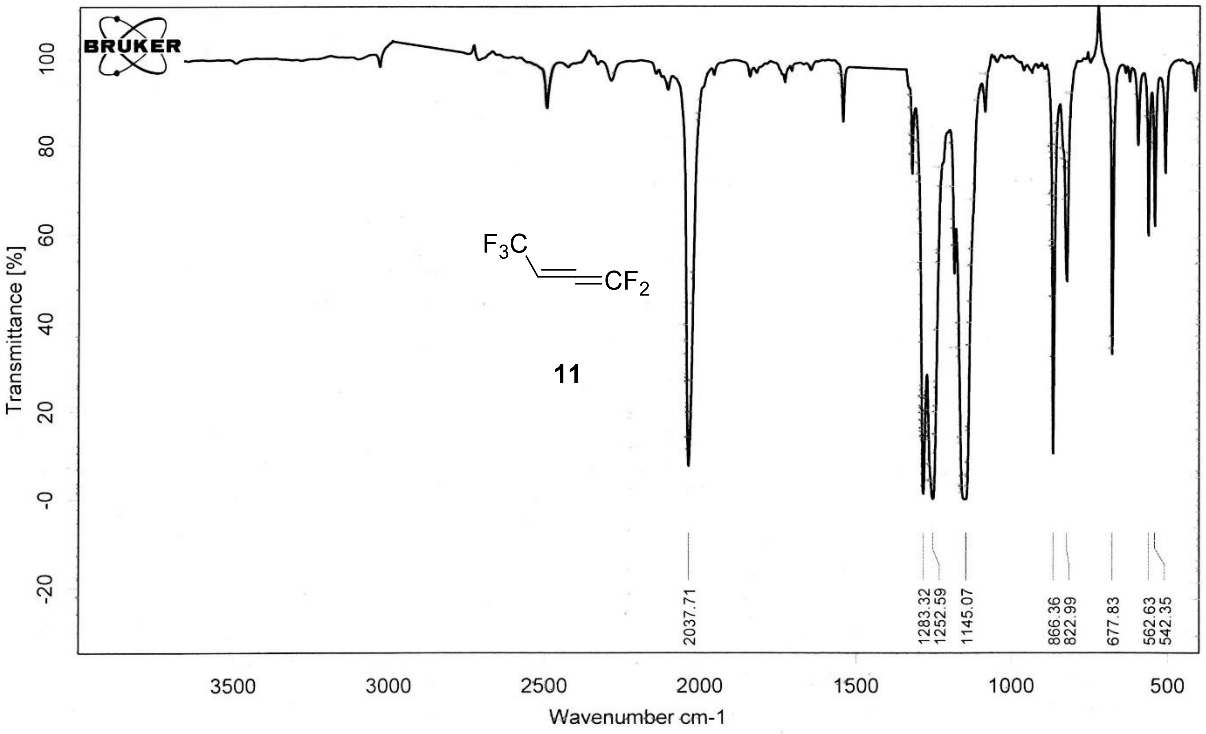

Figure S24. IR spectrum (neat) of **11**.

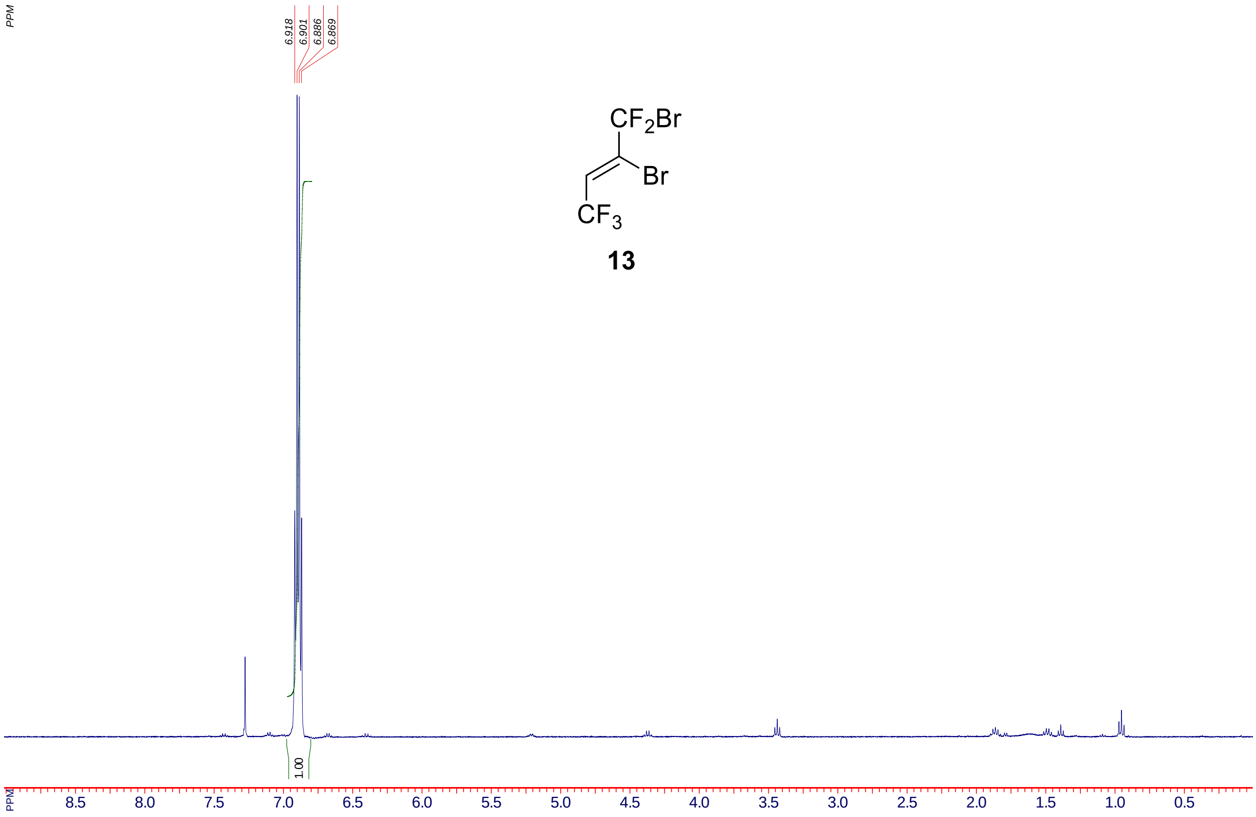

Figure S25. ^{1}H NMR of **13** ($CDCl_3$, 400 MHz).

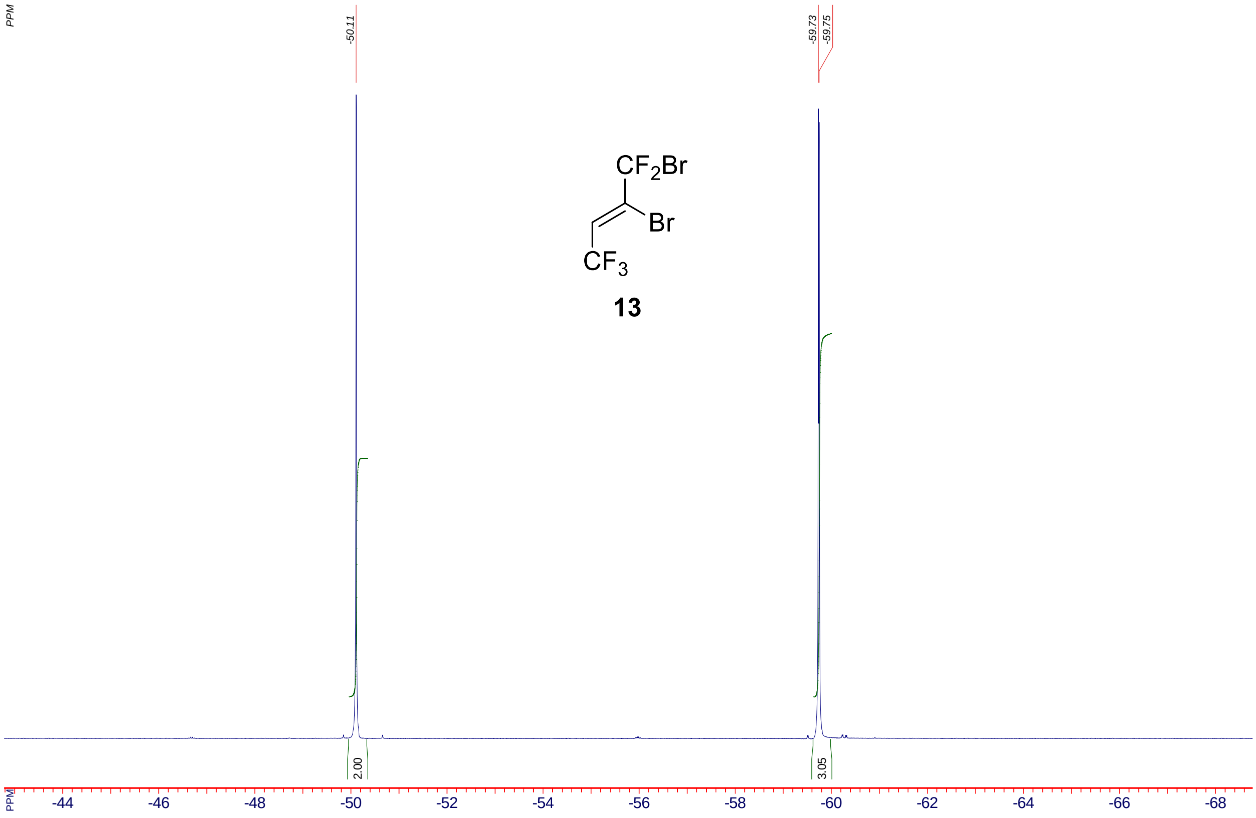

Figure S26. ^{19}F NMR of **13** ($CDCl_3$, 376.5 MHz).

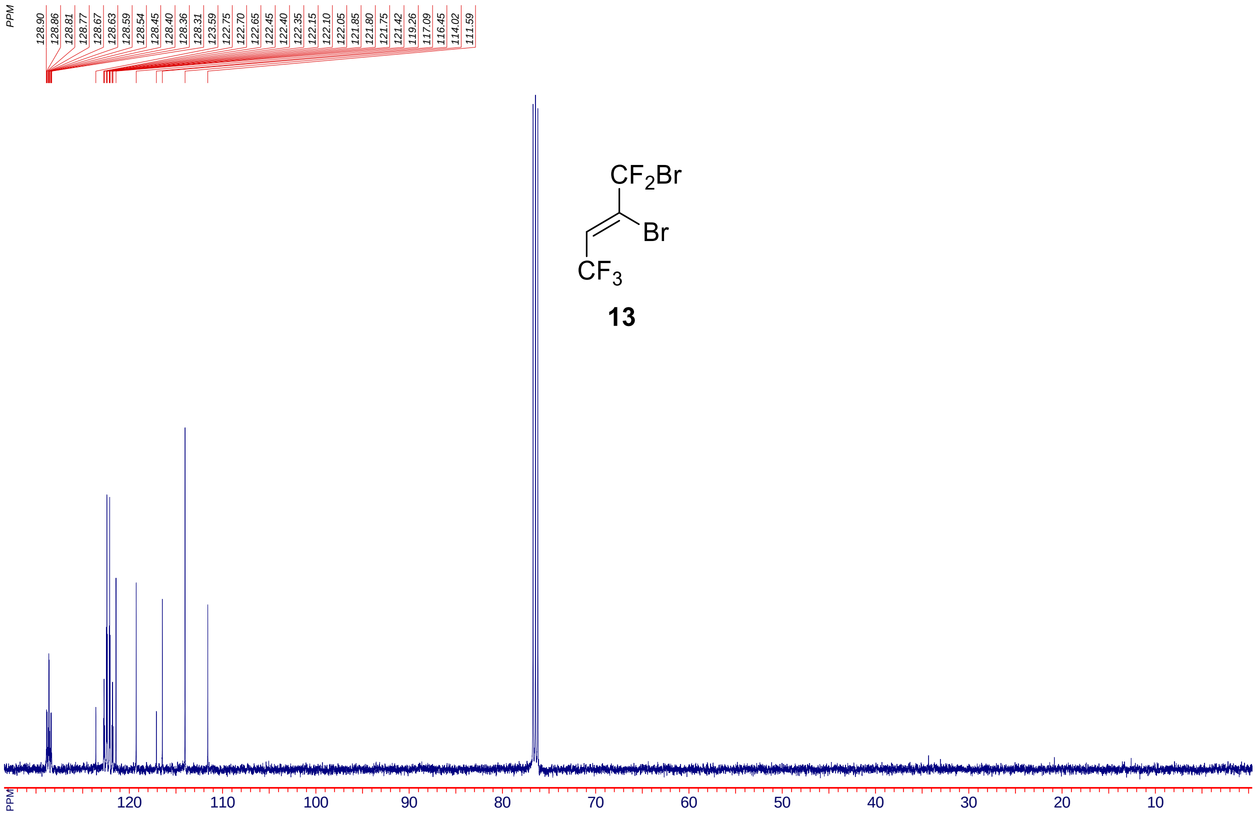

Figure S27. ^{13}C NMR of **13** ($CDCl_3$, 125.67 MHz).

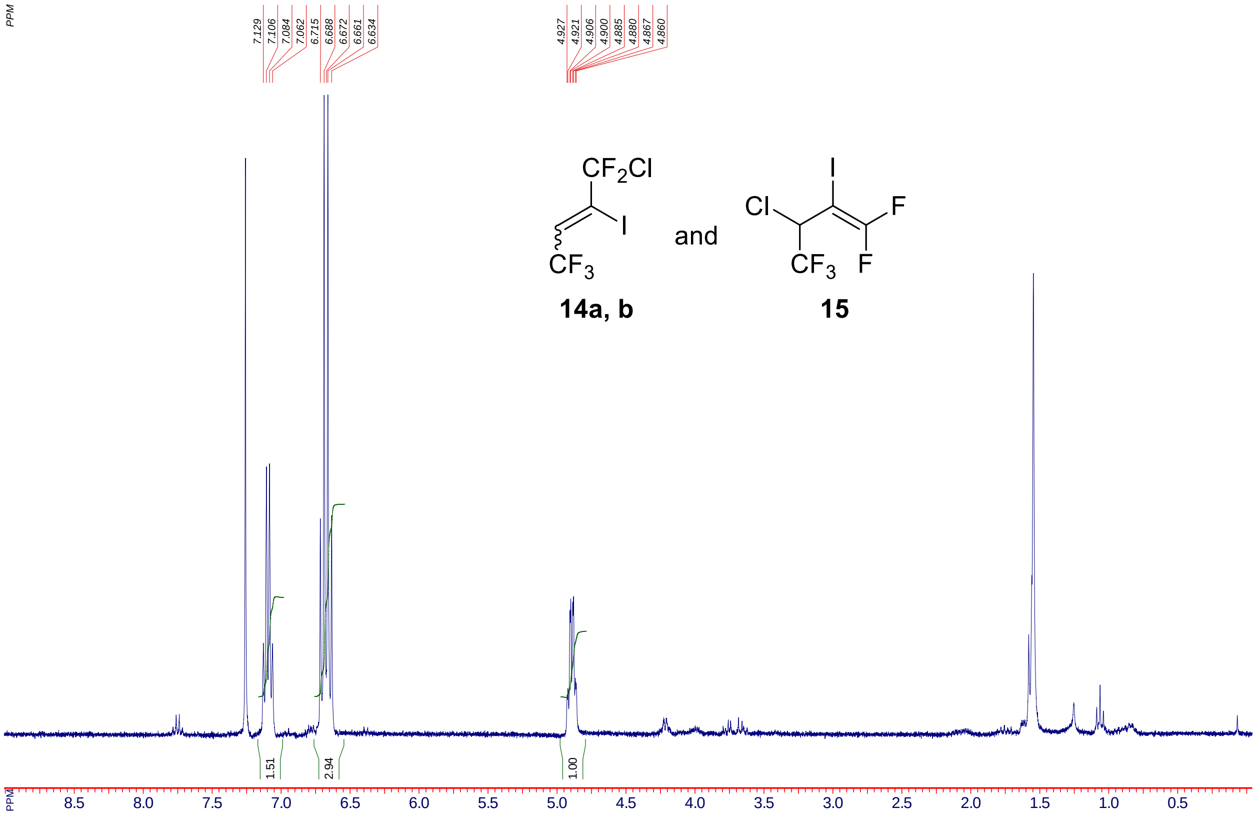

Figure S28. ^{1}H NMR of **14a, b** and **15** ($CDCl_3$, 301.5 MHz).

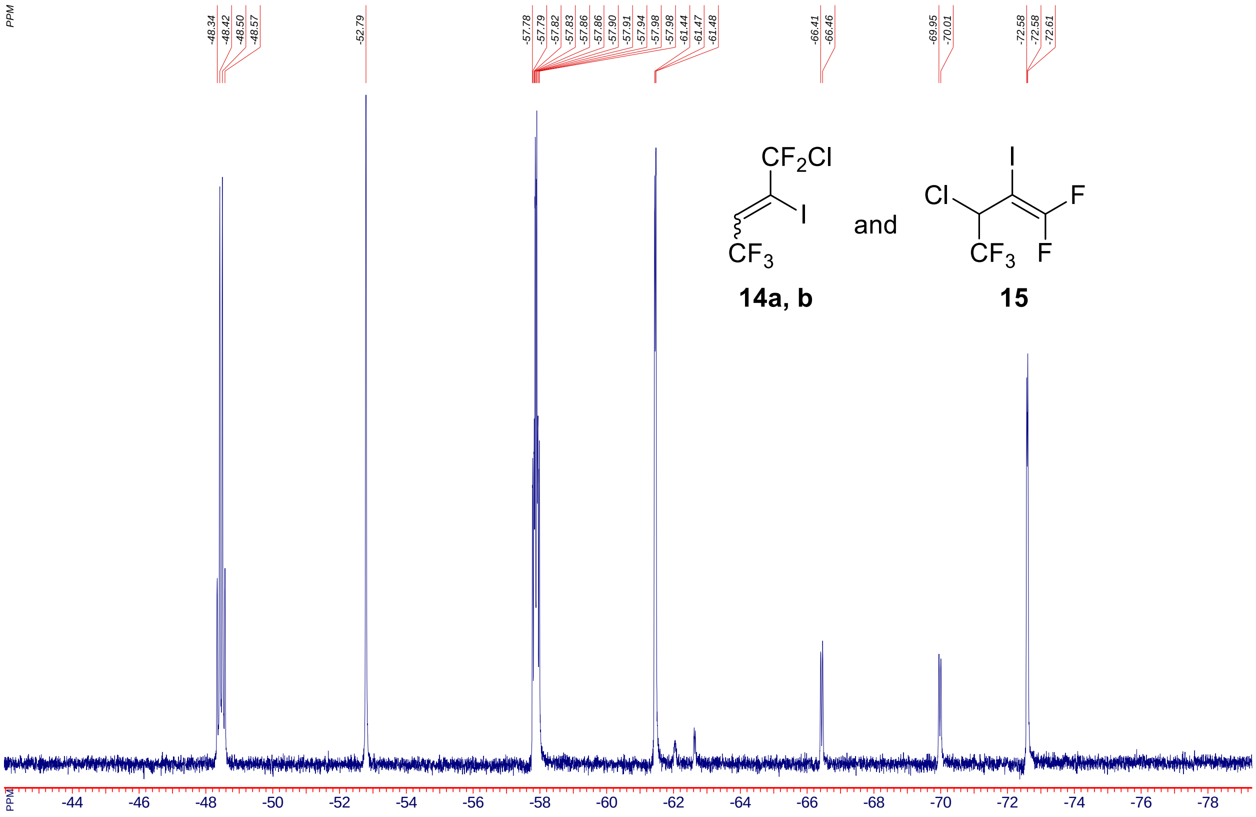

Figure S29. ^{19}F NMR of **14a, b** and **15** ($CDCl_3$, 188.14 MHz).

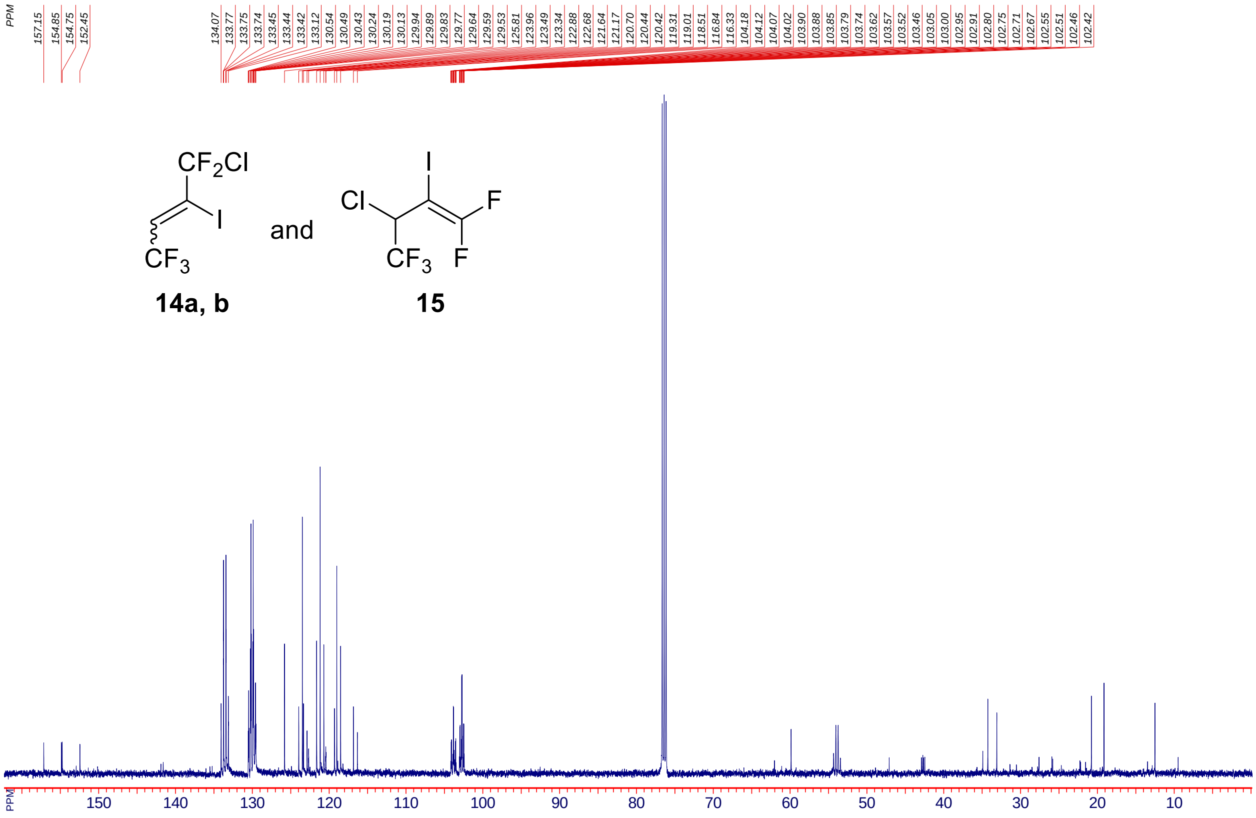

Figure S30. ^{13}C NMR of **14a, b** and **15** ($CDCl_3$, 125.67 MHz).

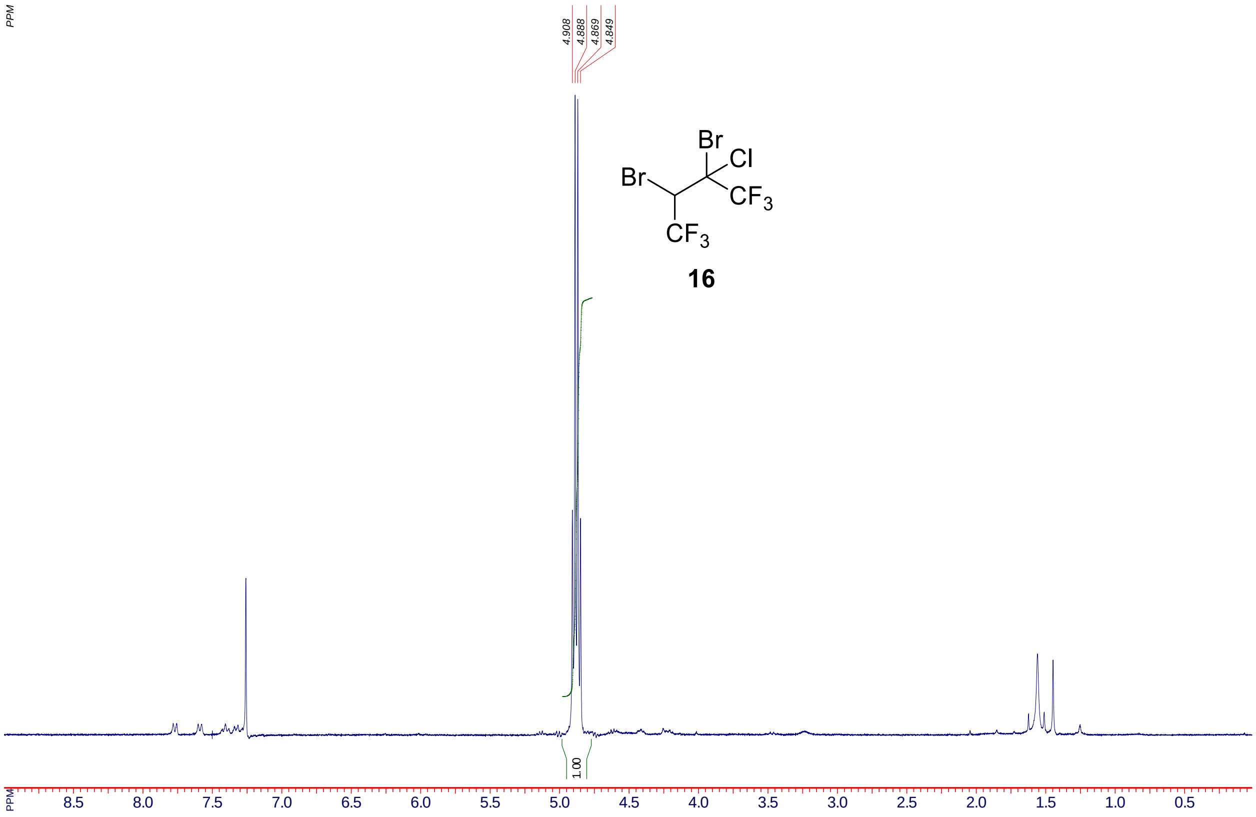

Figure S31. ^{1}H NMR of **16** ($CDCl_3$, 400 MHz).

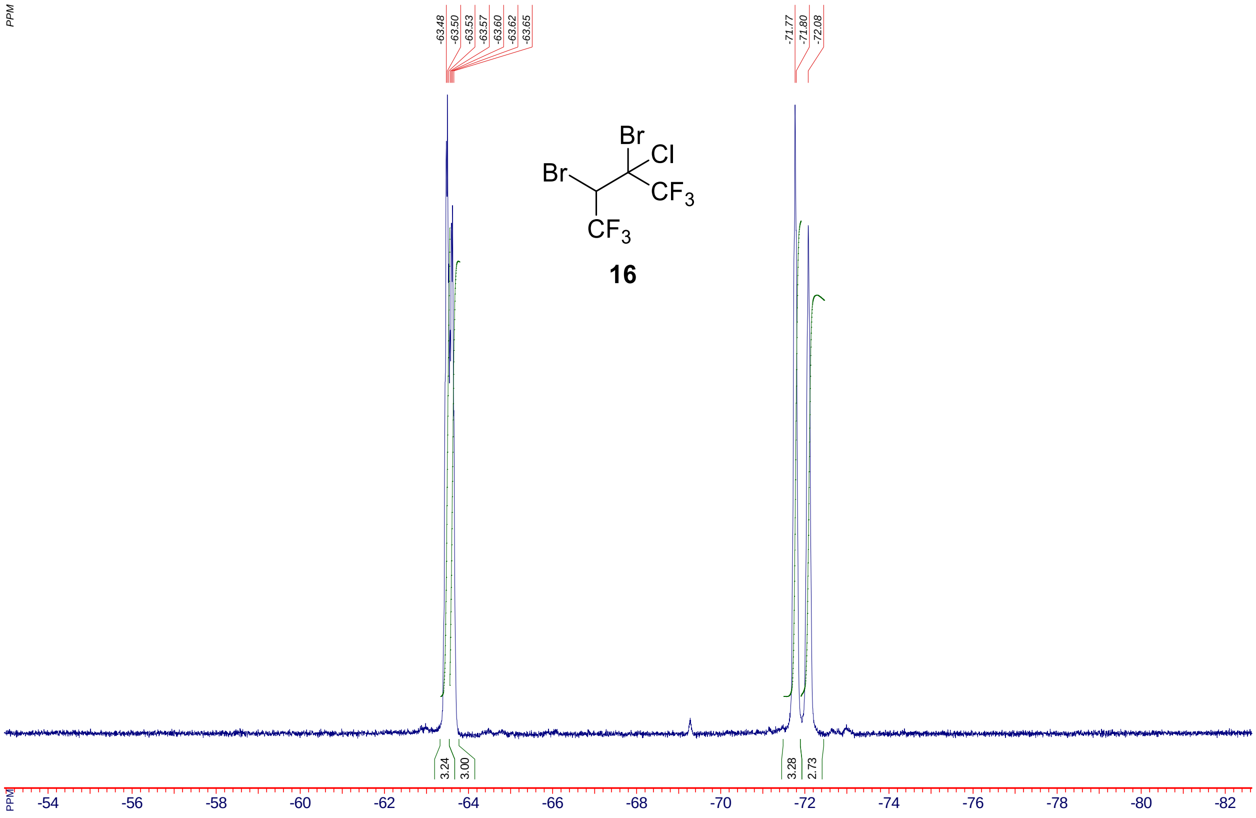

Figure S32. ^{19}F NMR of **16** ($CDCl_3$, 188.14 MHz).

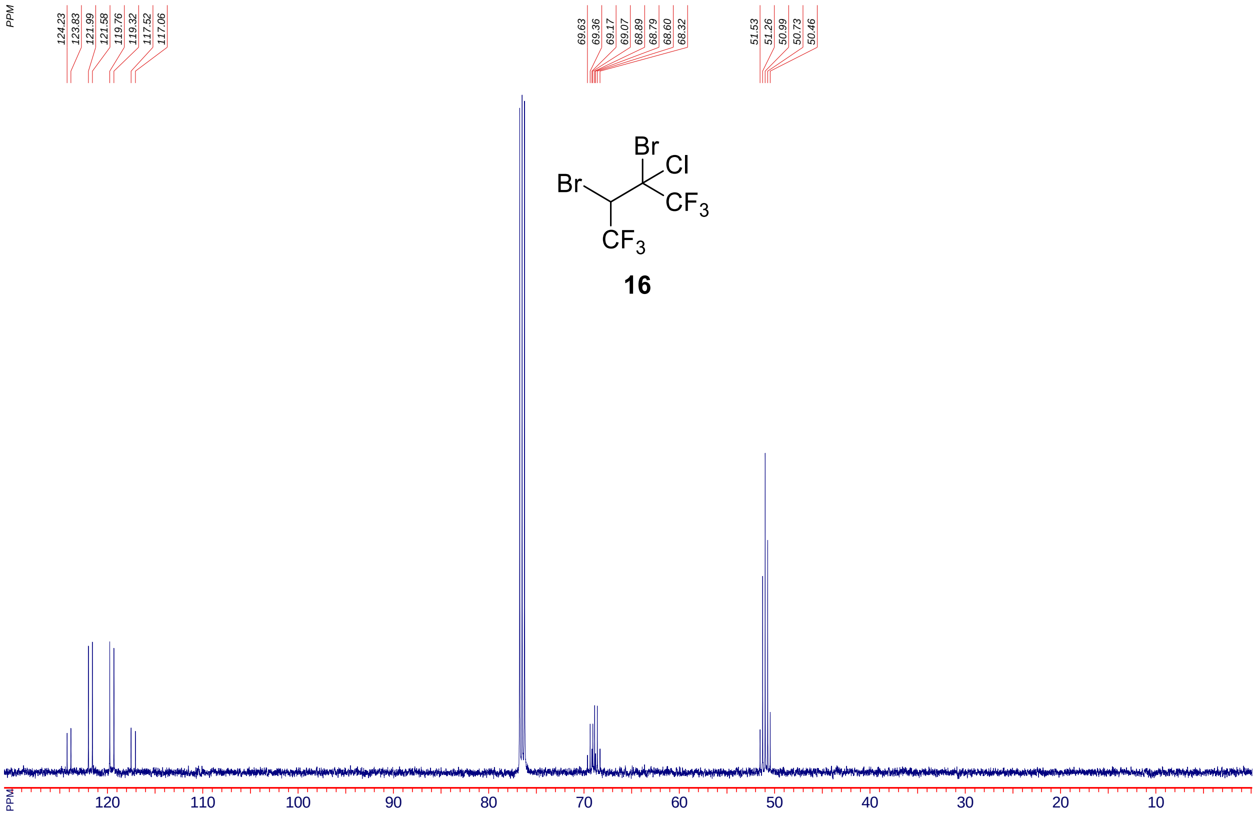

Figure S33. ^{13}C NMR of **16** ($CDCl_3$, 125.67 MHz).

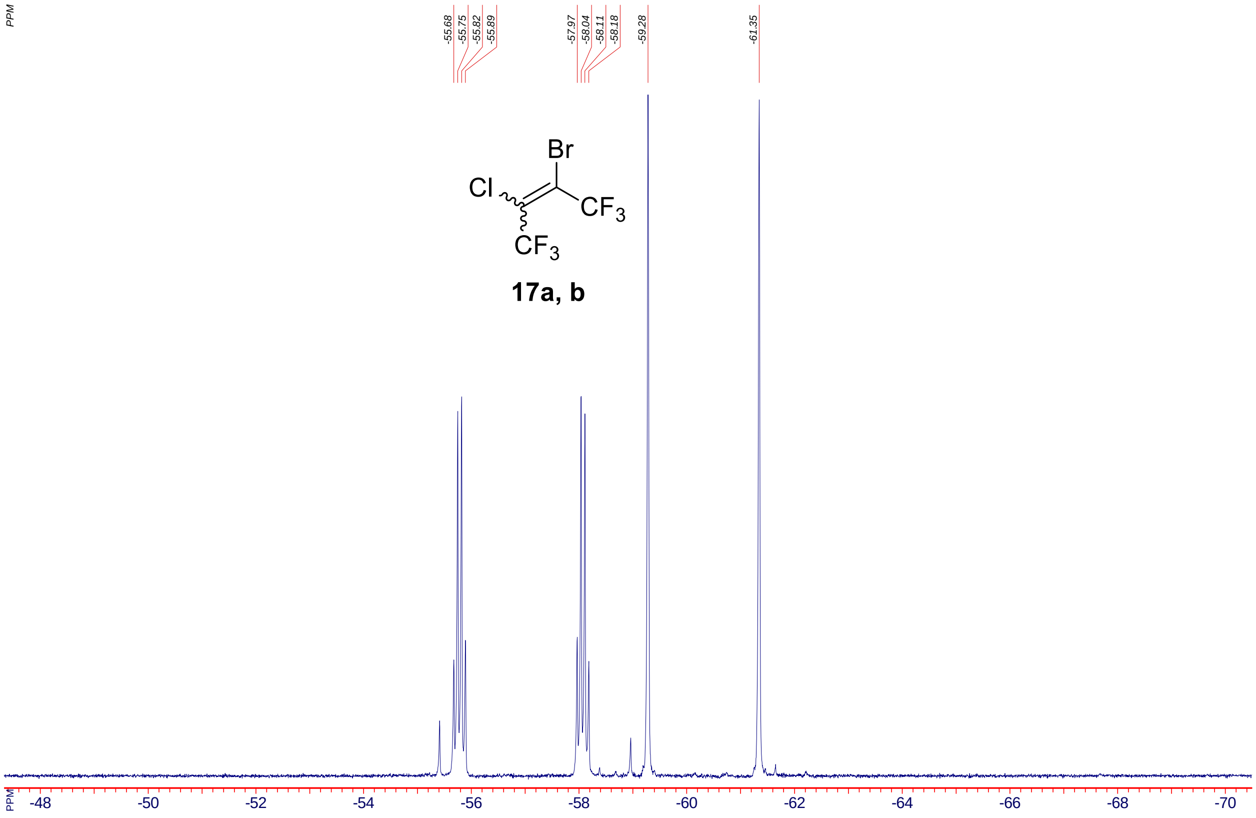

Figure S34. ^{19}F NMR of **17a, b** ($CDCl_3$, 188.14 MHz).

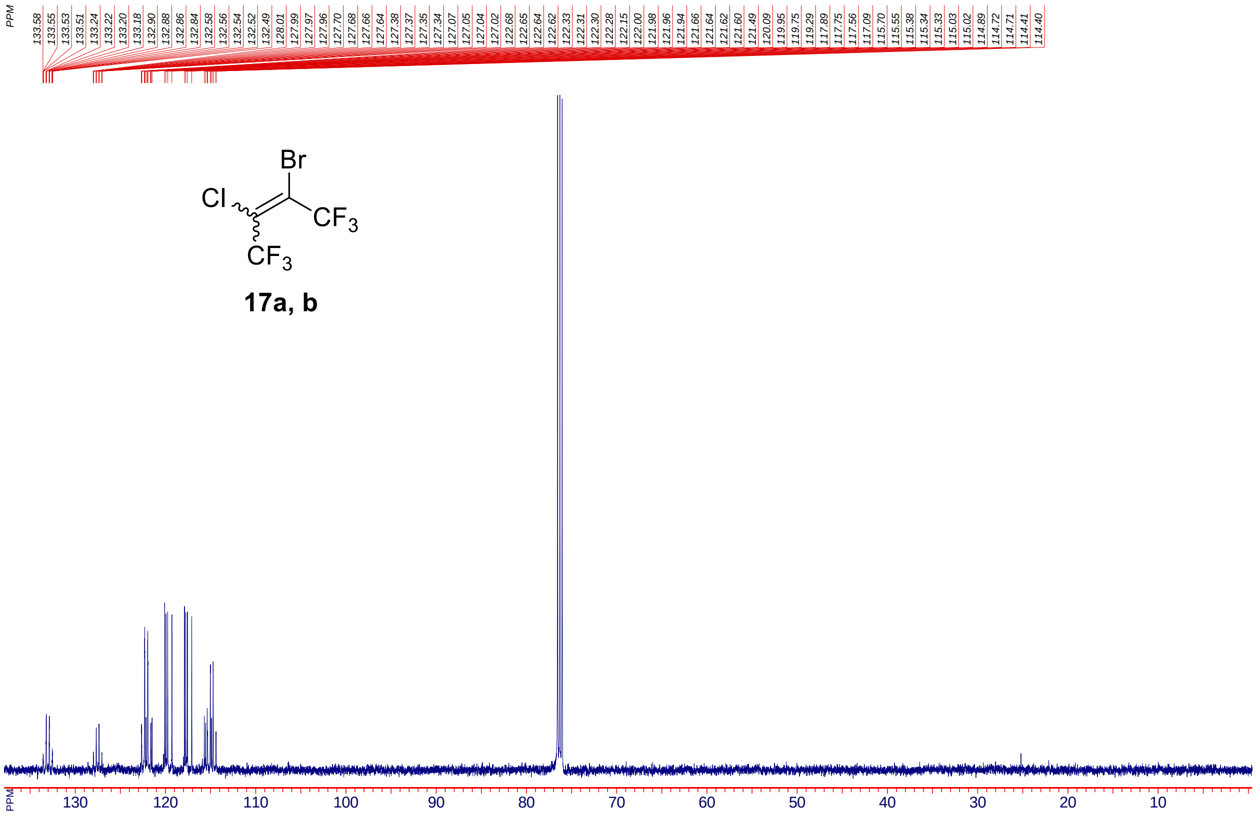

Figure S35. ^{13}C NMR of **17a, b** ($CDCl_3$, 125.67 MHz).

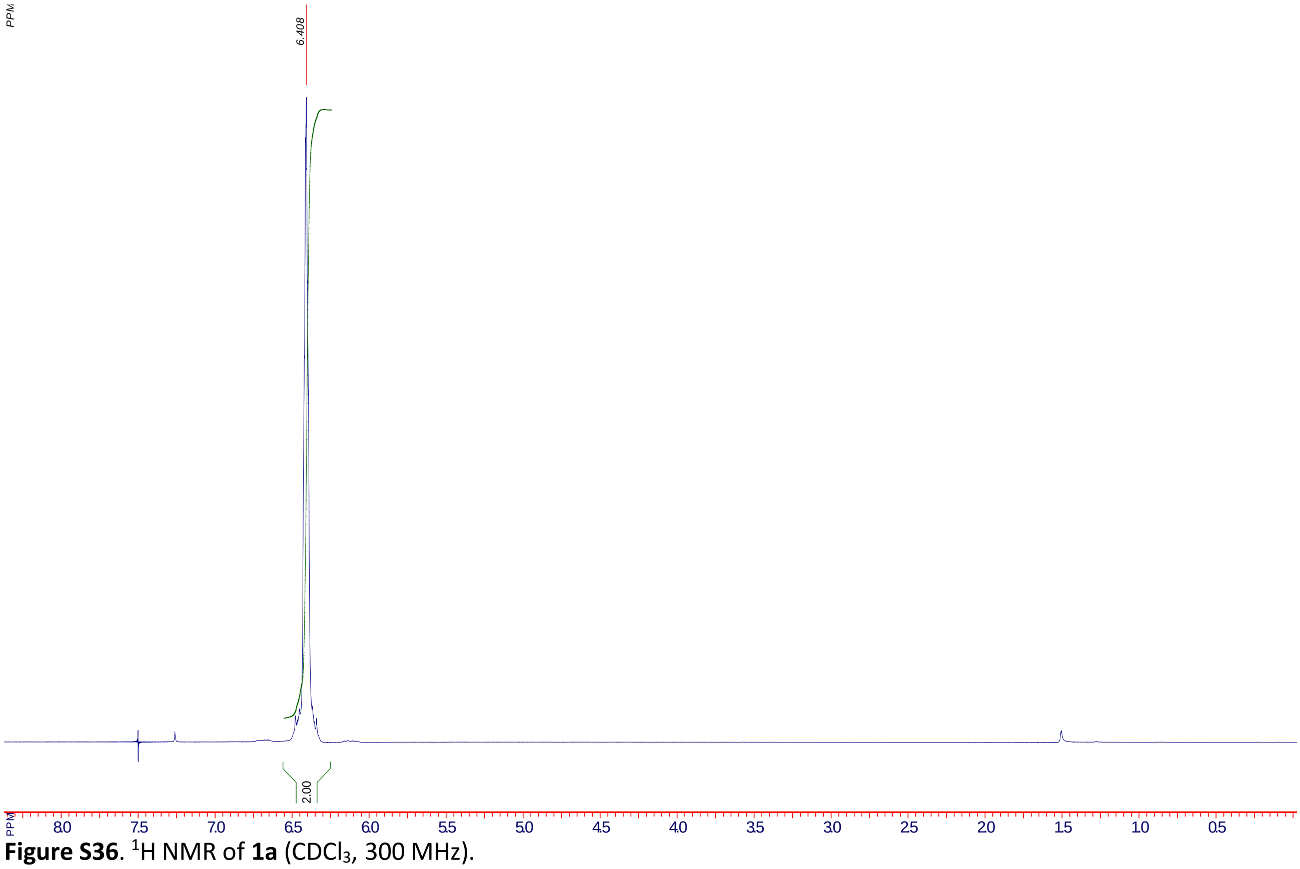

Figure S36. ^{1}H NMR of **1a** ($CDCl_3$, 300 MHz).

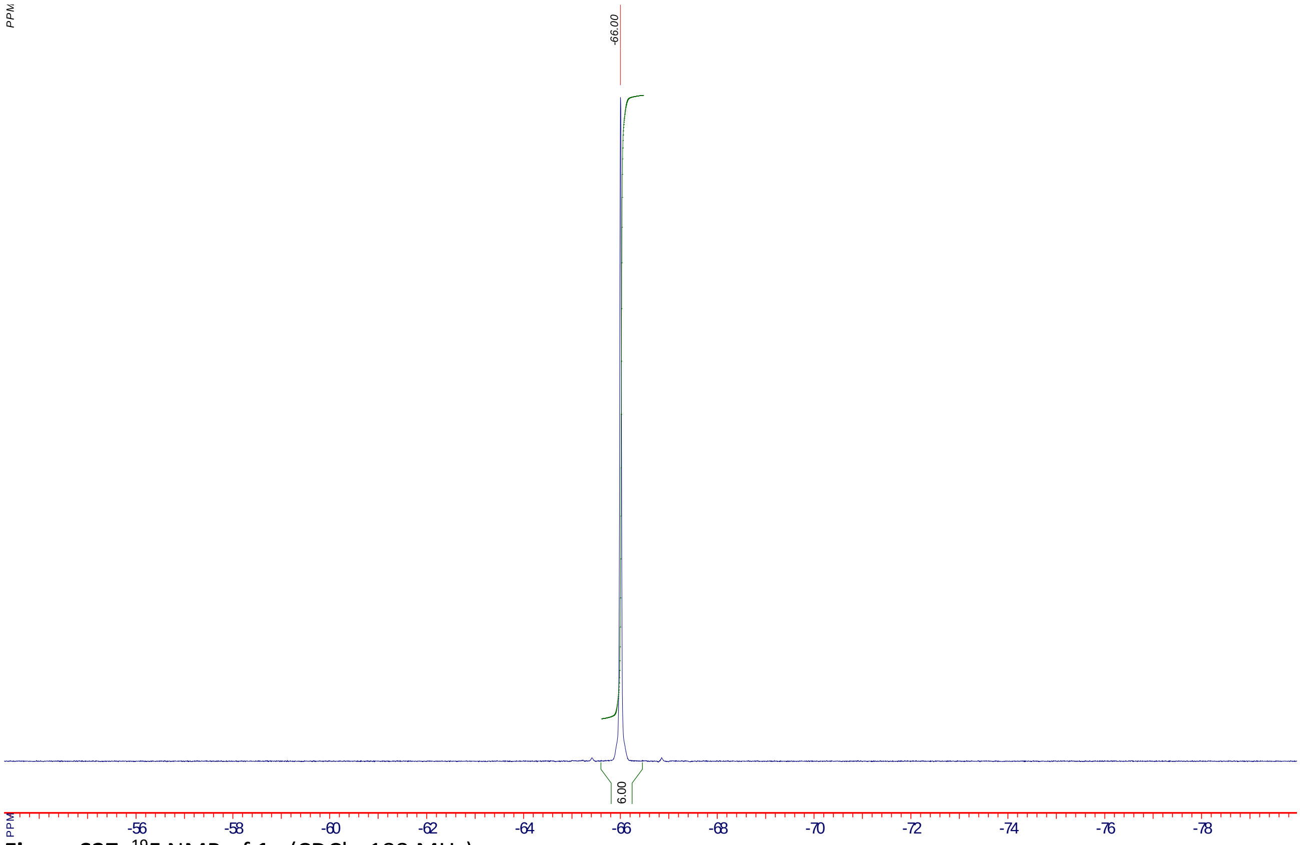

Figure S37. ^{19}F NMR of **1a** ($CDCl_3$, 188 MHz).

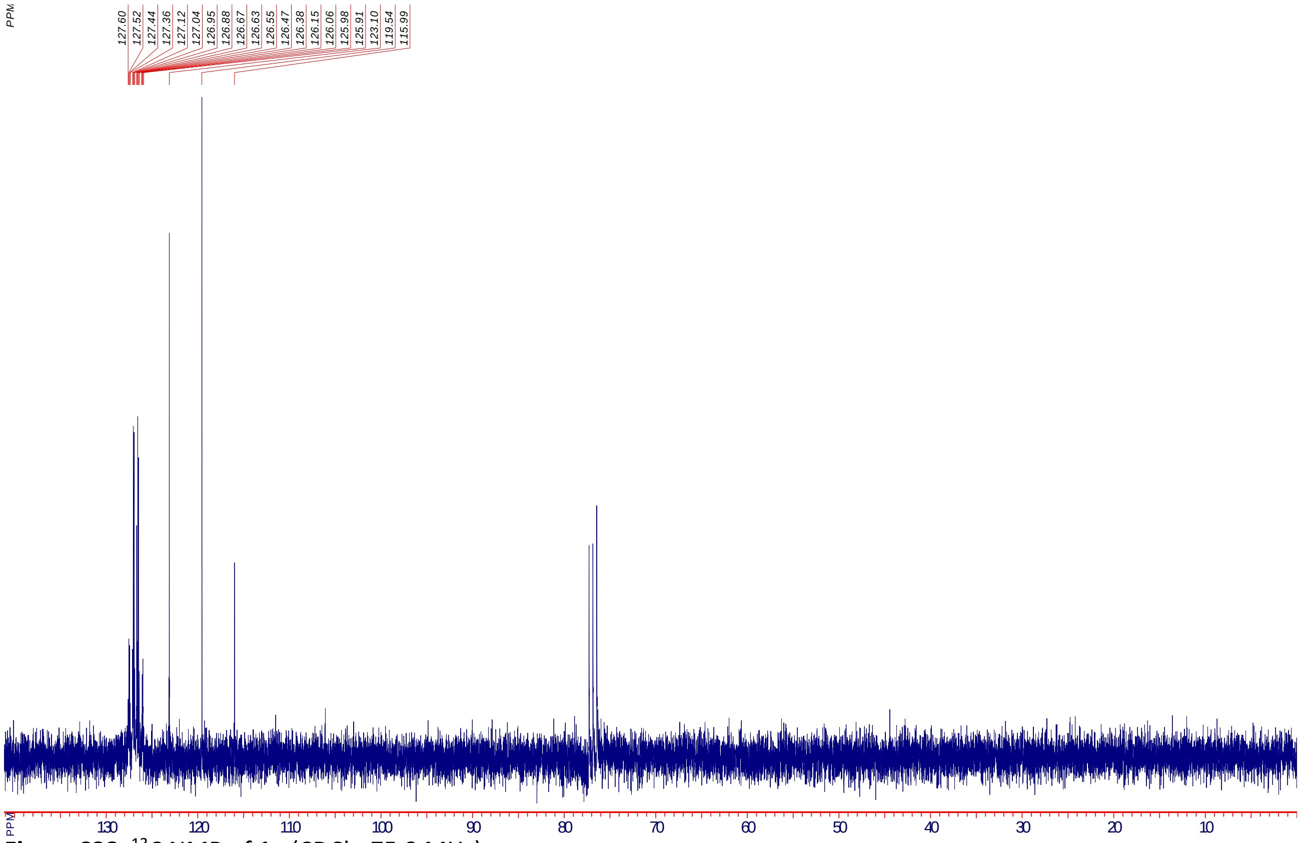

Figure S38. ^{13}C NMR of **1a** ($CDCl_3$, 75.8 MHz).

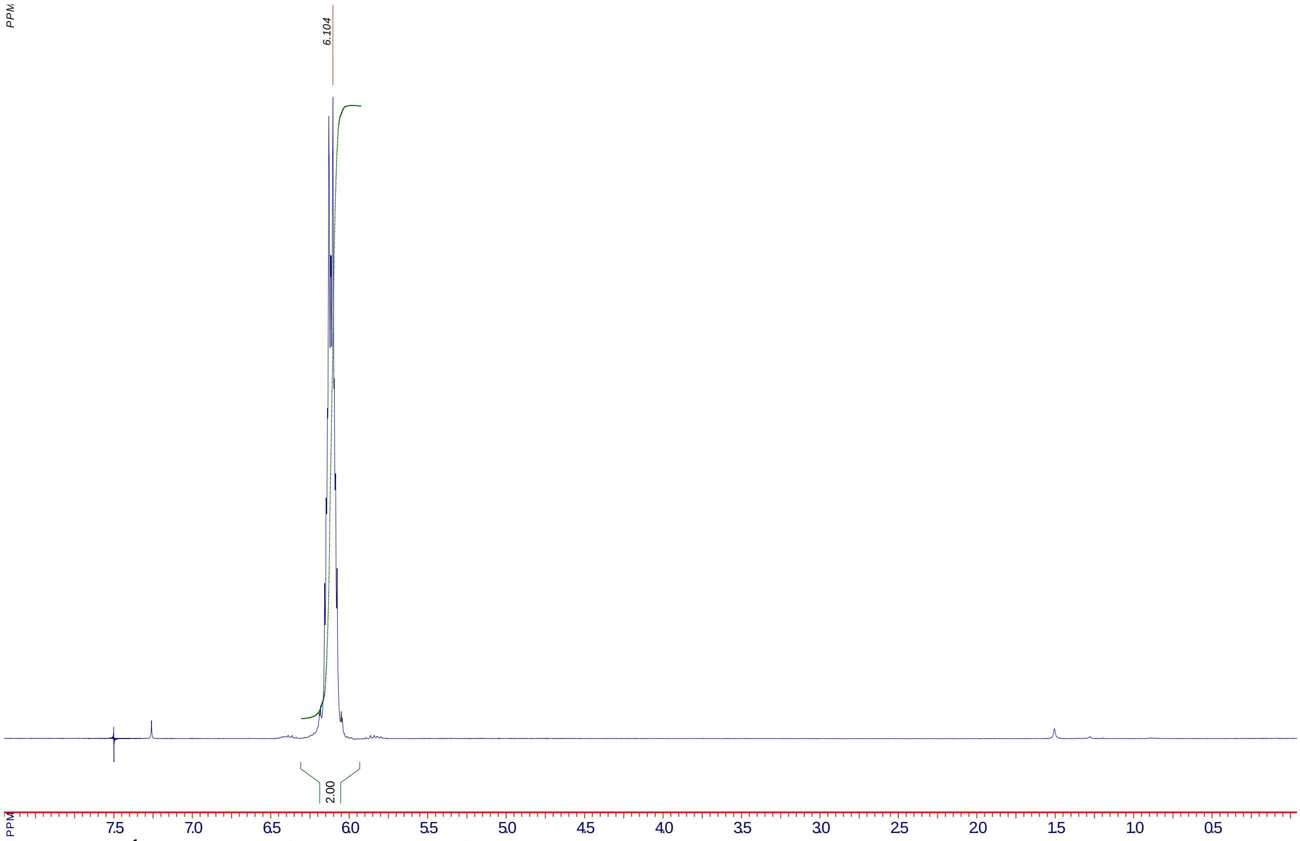

Figure S39. ^{1}H NMR of **1b** ($CDCl_3$, 300 MHz).

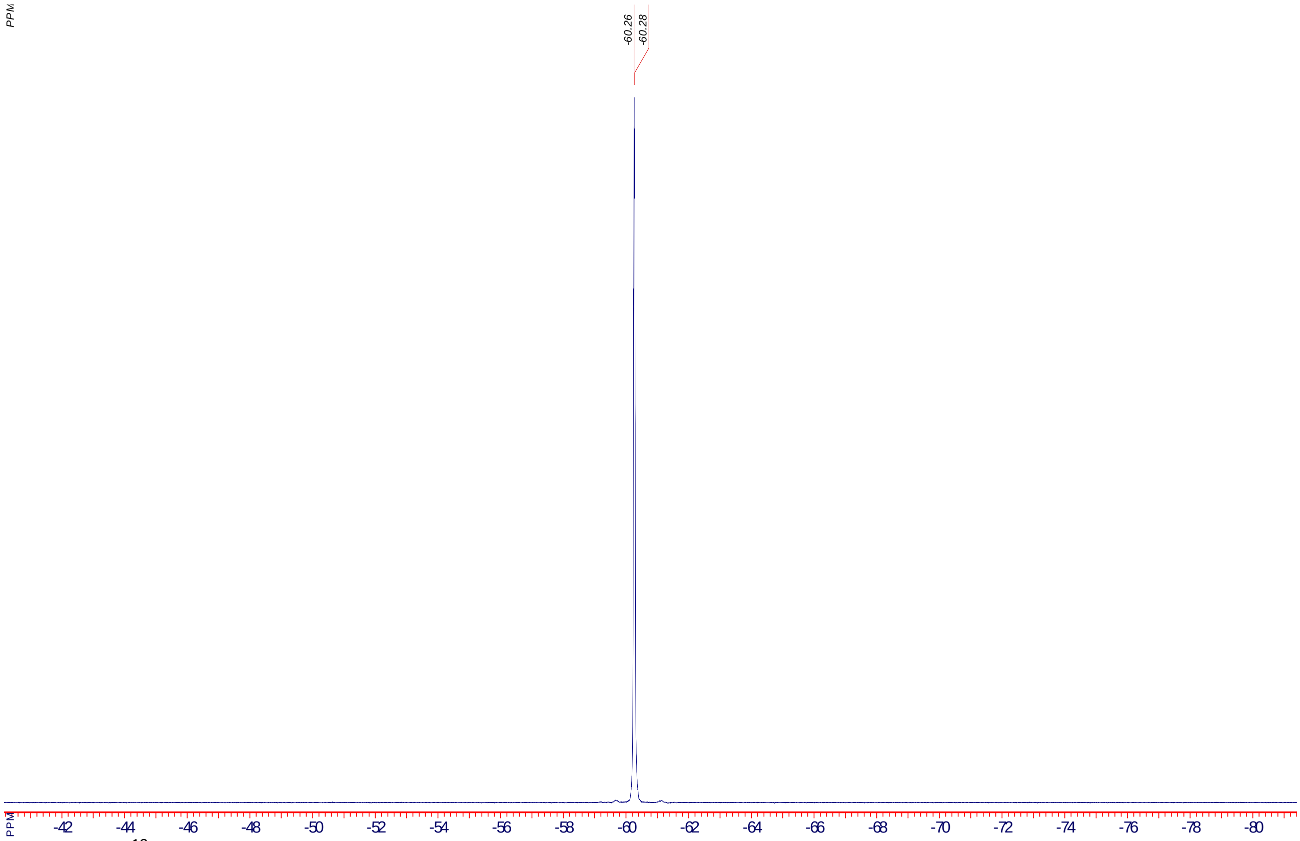

Figure S40. ^{19}F NMR of **1b** ($CDCl_3$, 188 MHz).

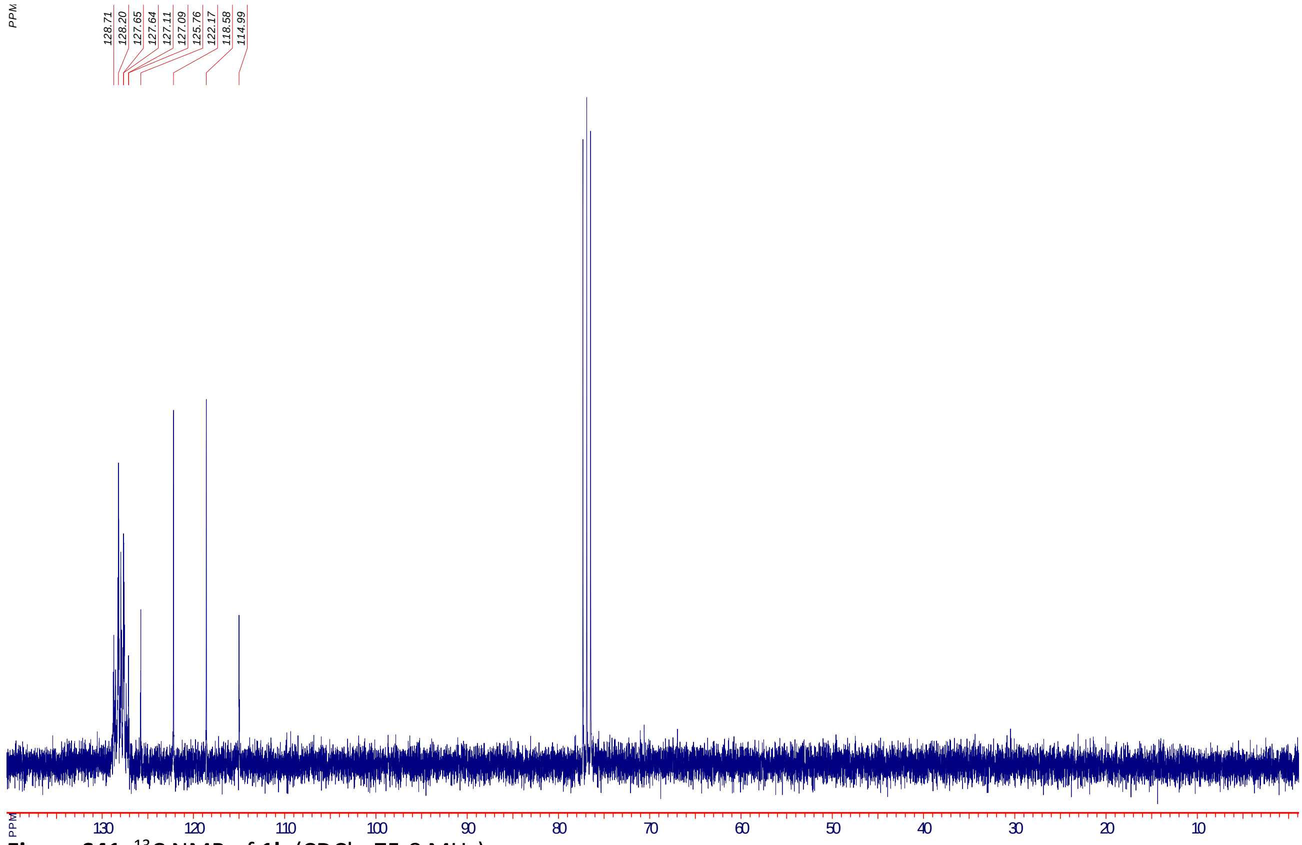

Figure S41. ^{13}C NMR of **1b** ($CDCl_3$, 75.8 MHz).

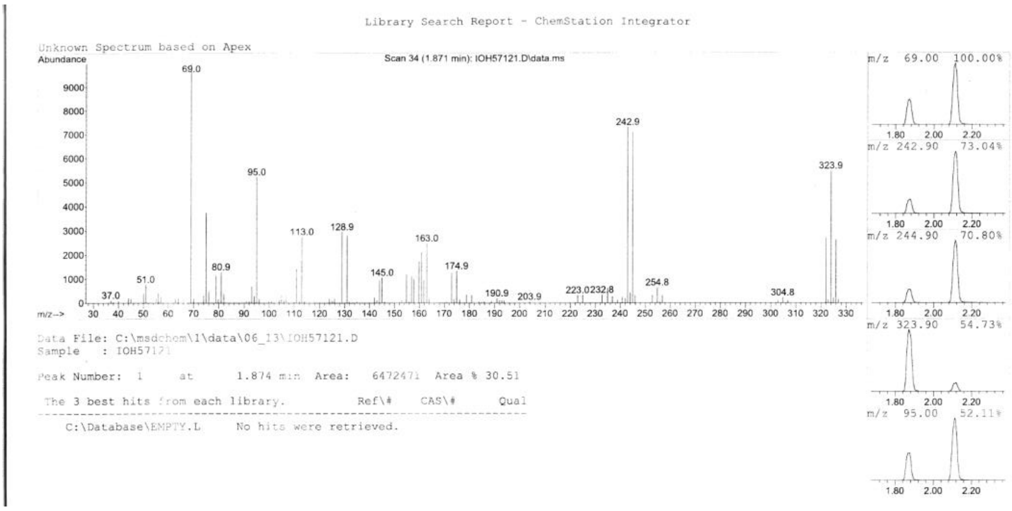

Figure S42. Mass-spectrum of **2**

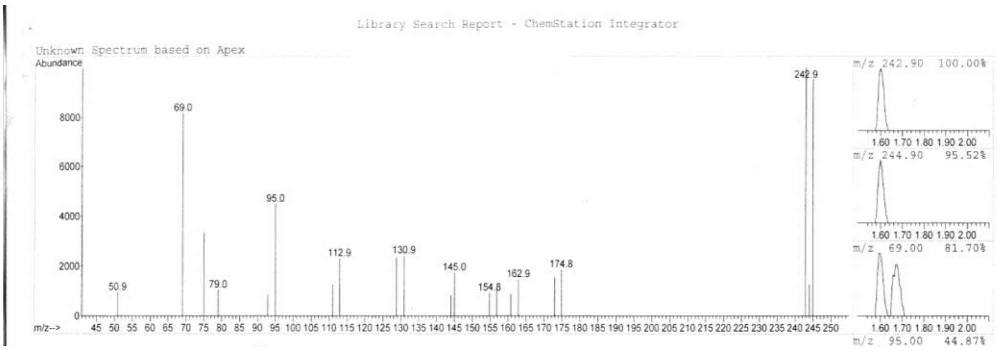

Figure S43. Mass-spectrum of **3a,b**

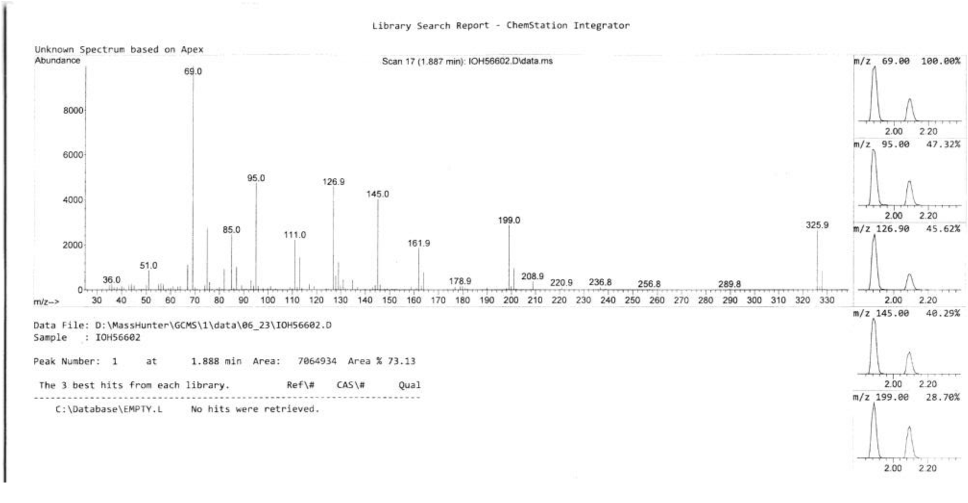

Figure S44. Mass-spectrum of **5**

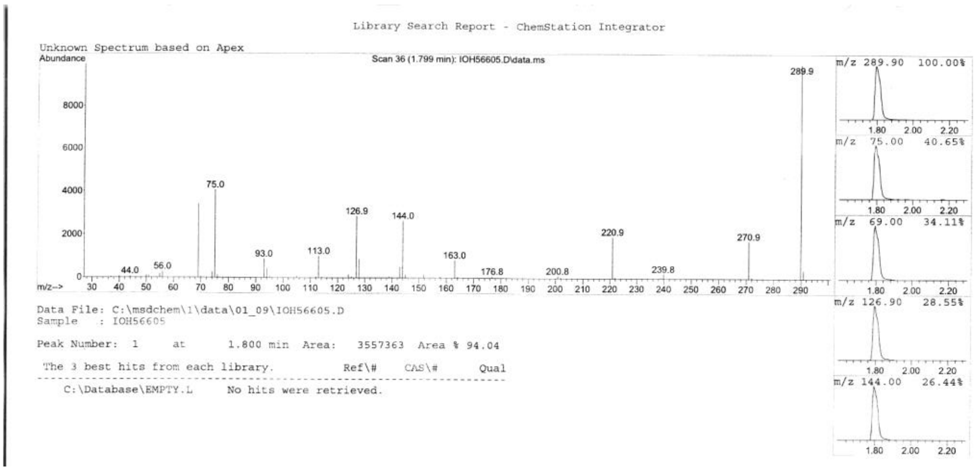

Figure S45. Mass-spectrum of **7a,b**

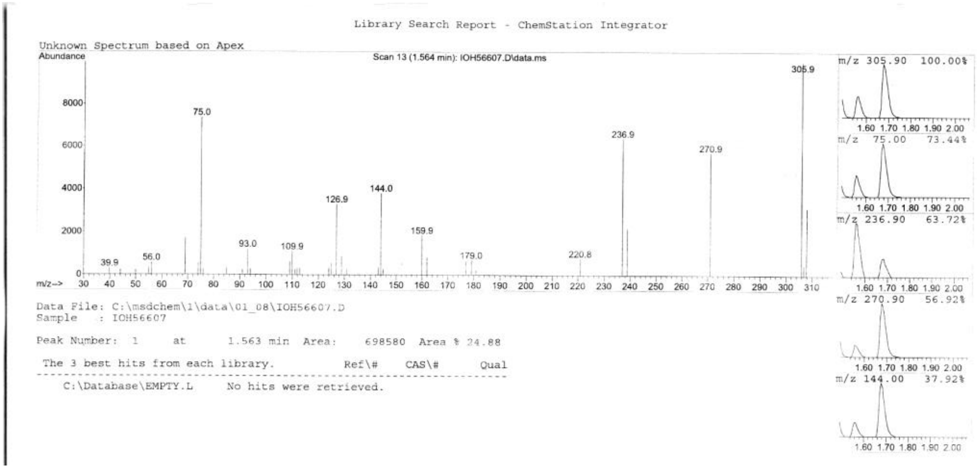

Figure S46. Mass-spectrum of **13**

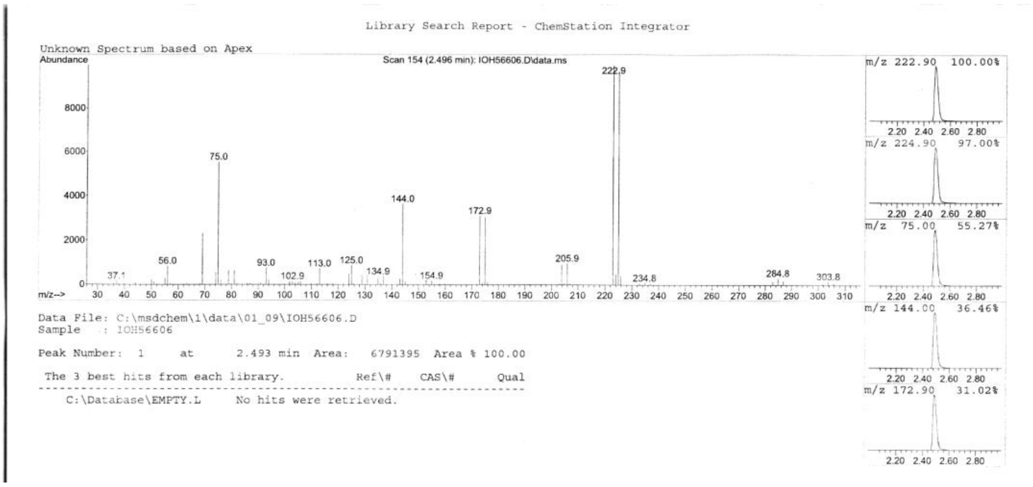

Figure S47. Mass-spectrum of **14a,b, 15**

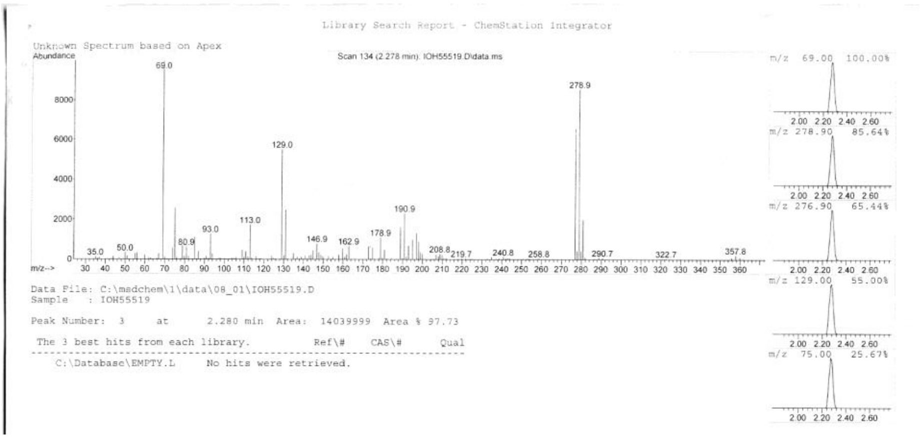

Figure S48. Mass-spectrum of **16**

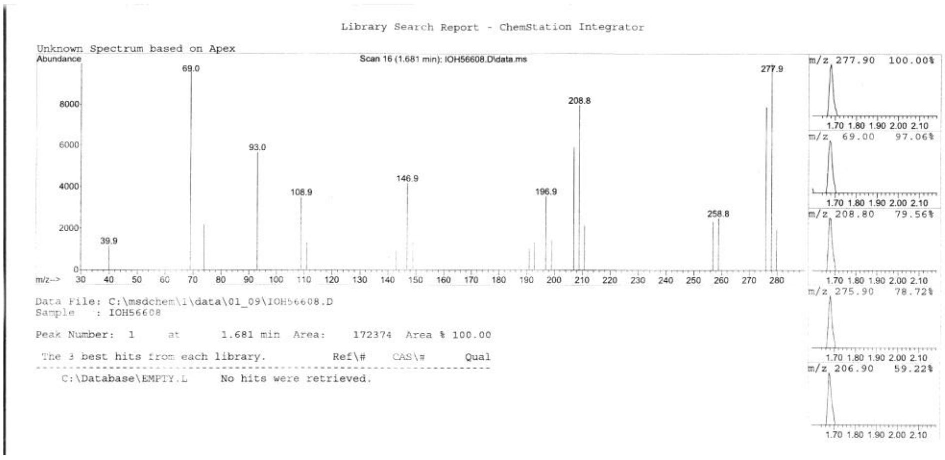

Figure S49. Mass-spectrum of **17**

3. References

1. Bo; Zh.; Jian, L.; Wei, Zh.; Jijun, Z.; Sheng, H.; Zhiqiang, Y.; Xiaobo, T.; Zhijun, H.; Jianping, K.; Fengxian, L. Catalytic conversion method for hexachlorobutadiene. *CN. Patent* 110372471, October 25, **2019**.

2. Jian, L.; Bo, Zh., Wei, Zh.; Jijun, Z.; Sheng, H.; Zhiqiang, Y.; Xiaobo, T.; Zhijun, H.; Jianping, K.; Fengxian, L. Synthesis method of 2-chloro-1,1,1,4,4,4-hexafluoro-2-butene. *CN. Patent* 110372472, October 25, **2019**.

3. Kirij, N.V.; Filatov, A.A.; Yagupolskii, Yu.L.; Peng, S.; Jackson, A. *J. Fluorine Chem.* **2022**, *253*, 109922. https://doi.org/10.1016/j.jfluchem.2021.109922
